# Priorisierung von Forschungsfragen in Gesundheitskrisen – Vorstellung eines Konzeptes, entwickelt in der COVID-19 Pandemie

**DOI:** 10.1007/s00103-024-03985-4

**Published:** 2024-12-05

**Authors:** Annika Ziegler, Angela M. Kunzler, Sebastian Voigt-Radloff, Jochen Schmitt, Onnen Moerer, Simone Scheithauer, Heike Heytens, Christian Apfelbacher, Joerg J. Meerpohl

**Affiliations:** 1 Institut für Evidenz in der Medizin, Universitätsklinikum Freiburg, Medizinische Fakultät, Albert-Ludwigs-Universität Freiburg, Freiburg, Deutschland; 2 Institut für Medizinische Biometrie und Statistik, Universitätsklinikum Freiburg, Medizinische Fakultät, Albert-Ludwigs-Universität Freiburg, Freiburg, Deutschland; 3 Zentrum für Evidenzbasierte Gesundheitsversorgung (ZEGV), Medizinische Fakultät und Universitätsklinikum Carl Gustav Carus, Technische Universität Dresden, Dresden, Deutschland; 4 Klinik für Anästhesiologie, Universitätsmedizin Göttingen, Georg-August-Universität Göttingen, Göttingen, Deutschland; 5 Institut für Krankenhaushygiene und Infektiologie, Universitätsmedizin Göttingen, Georg-August-Universität Göttingen, Göttingen, Deutschland; 6 Institut für Sozialmedizin und Gesundheitssystemforschung, Universitätsklinikum Magdeburg, Medizinische Fakultät, Otto-von-Guericke-Universität Magdeburg, Magdeburg, Deutschland; 7 Cochrane Deutschland, Cochrane Deutschland Stiftung, Freiburg, Deutschland

**Keywords:** Forschungspriorisierung, Konzeptpapier, Netzwerk Universitätsmedizin (NUM), Ressourcenallokation, Krisenmanagement, Research prioritization, Concept paper, Network University Medicine (NUM), Resource allocation, Crisis management

## Abstract

In akuten Krisen wie der COVID-19-Pandemie müssen wissenschaftliche Fragestellungen schnell bearbeitet werden, um die Gesundheit der Bevölkerung zu schützen und die Funktion des Gesundheitssystems aufrechtzuerhalten. Die vorherrschende Dringlichkeit und die Vielzahl zu bearbeitender Fragestellungen bei gleichzeitiger Limitation von zeitlichen, personellen oder monetären Ressourcen machen eine Priorisierung unabdingbar. Im Projekt *COVID-19-Evidenz-Oekosystem *(CEOsys) des *Netzwerks Universitätsmedizin* (NUM) wurde ein Verfahren zur schnellen Priorisierung von Forschungsfragen speziell für Evidenzsynthesen und klinische Leitlinienempfehlungen initiiert, das im Folgeprojekt *PREparedness and Pandemic Response in Deutschland* (PREPARED) weiterentwickelt wurde.

Entstanden ist das „Konzeptpapier Forschungspriorisierung“, das eine generischere Ausrichtung hat. Die Inhalte des Konzepts werden in diesem Beitrag dargestellt. Kernthemen sind einerseits grundsätzliche Prinzipien einer gelingenden Priorisierung sowie ein konkreter 7‑schrittiger Prozess mit Angaben zu organisatorischen Rahmenbedingungen und zum Ablauf. Das Konzept bietet Adaptionsmöglichkeiten, da Forschungspriorisierung stark kontextabhängig ist.

Die Anwendung eines solchen systematischen, transparenten Priorisierungsprozesses trägt dazu bei, nachvollziehbare und informierte Entscheidungen darüber zu treffen, welche wissenschaftlichen Fragen Relevanz und Dringlichkeit aufweisen, in welcher Bearbeitungsreihenfolge sie stehen sollten und welche Fragestellungen nicht oder zu einem späteren Zeitpunkt bearbeitet werden müssen.

**Zusatzmaterial online:**

Zusätzliche Informationen sind in der Online-Version dieses Artikels (10.1007/s00103-024-03985-4) enthalten.

## Abstract (en)

In acute crises such as the COVID-19 pandemic, scientific questions need to be addressed quickly in order to protect the health of the population and to maintain the function of the healthcare system. The prevailing urgency and the large number of issues to be addressed, combined with the limitation of time, personnel, or monetary resources make prioritization indispensable. In the COVID-19 Evidence Ecosystem (CEOsys) project initiated by the University Medicine Network (NUM), a procedure for the rapid prioritization of questions was used specifically for evidence syntheses and clinical guideline recommendations, which was further developed in the follow-up project PREparedness and Pandemic Response in Germany (PREPARED).

The result is a concept paper on the prioritization of research questions and topics with a more generic orientation. The content of the concept is presented in this article. The core subjects are basic principles of successful prioritization as well as an explicit seven-step process with information on organizational framework conditions and the procedure. The concept offers possibilities for adaptation, as research prioritization is highly context-dependent.

The application of such a systematic, transparent prioritization process contributes to comprehensible and informed decisions about which research questions are relevant and urgent, in which order they should be processed, and which issues are not critically urgent or have to be postponed.

## Hintergrund

Die Relevanz der Priorisierung von Gesundheitsforschung (engl. „research prioritization“ oder „research priority setting“) spiegelt sich bereits seit mehr als 20 Jahren in der internationalen Literatur wider [1]. Denn begrenzte Ressourcen wie Zeit, Personal oder finanzielle Mittel müssen adäquat eingesetzt werden. Wissenschaftler:innen, aber auch weitere Entscheidungsträger:innen stehen vor der Herausforderung Forschungsinhalte festlegen zu müssen. Für diese Forschungspriorisierung gibt es verschiedene Ansätze und Konzepte in der Literatur [2–5].

Während der COVID-19-Pandemie als akuter Gesundheitskrise zeigte sich ein dringender Forschungsbedarf, der sich über viele medizinische Fachdisziplinen erstreckte und maßgeblich das Sicherstellen der öffentlichen Gesundheit (Public Health) zum Ziel hatte. Der Priorisierung von Forschung kommt in einer solch akuten Bedarfslage eine noch bedeutendere Rolle zu, weil Ressourcen unter großem Zeitdruck zielgerichtet verteilt werden müssen [6].

Im ersten Jahr der COVID-19-Pandemie (2020) wurde mit dem neu gegründeten Netzwerk Universitätsmedizin (NUM)^1^ ein Zusammenschluss der deutschen Universitätskliniken zur COVID-19-bezogenen Forschung und für den Aufbau dazugehöriger Forschungsinfrastrukturen initiiert. Im Projekt *COVID-19-Evidenz-Oekosystem (CEOsys)*^2^ der ersten Förderphase (2020–2021) wurden durch den zügigen Zusammenschluss mehrerer Standorte über 30 Evidenzsynthesen (z. B. systematische Übersichtsarbeiten, Scoping-Reviews) durchgeführt. Ziel von CEOsys war die Aufbereitung von Evidenz zu relevanten Forschungsfragen für Entscheidungstragende, insbesondere für die Etablierung von medizinischen Leitlinien. Aufgrund der Vielzahl an potenziellen Fragestellungen aus unterschiedlichen Fachdisziplinen wurde schnell deutlich, dass eine Bewertung der (zeitlichen) Dringlichkeit und aktuellen Relevanz erfolgen muss.

Der in CEOsys angewandte Priorisierungsprozess enthielt sowohl organisatorische als auch inhaltliche Komponenten, die in einem Ranking der Forschungsfragen und schließlich in einer Bearbeitungsreihenfolge mündeten. Er ist im Anhang 2 der Publikation zum Projekt CEOsys beschrieben und abgebildet [7]. Da im Jahr 2020 aufgrund der akuten gesundheitlichen Gefahrenlage wenig Zeit zur konzeptionellen Ausarbeitung und Optimierung des Prozesses blieb, fand im anschließenden NUM-Projekt *PREparedness and PAndemic REsponse in Deutschland (PREPARED)*^3^ eine Weiterentwicklung statt. PREPARED fokussierte den Aufbau von Strukturen zur Sicherstellung von politischer Handlungsfähigkeit im Pandemie- oder Krisenfall (Pandemic Preparedness). Aus CEOsys gewonnene Erkenntnisse über Barrieren für den Priorisierungsprozess wurden adressiert. Der Prozess zielt nun nicht mehr explizit auf die Priorisierung für Evidenzsynthesen und Leitlinienempfehlungen ab, sondern ist unabhängiger von der nachfolgenden Forschungsmethodik.

Das Ziel dieses Beitrags besteht darin, das entstandene Konzept zur Priorisierung von Forschungsfragen und -themen darzustellen, um es der interessierten Fachöffentlichkeit zugänglich zu machen. Dazu orientiert sich der Hauptteil des Beitrags am Aufbau des zugrunde liegenden Konzepts: Neben grundlegenden Prinzipien wird ein 7‑schrittiger Priorisierungsprozess vorgestellt. Im Fazit wird ein Ausblick auf weitere notwendige Schritte in der Weiterentwicklung und Etablierung von Priorisierungsansätzen im NUM und in der Gesundheitsforschung allgemein gegeben. Das ausführliche Konzeptpapier, inklusive beispielhafter Vorlagen für einzelne Schritte, ist als Onlinematerial beigefügt.

## Kontext: Forschungspriorisierung während der COVID-19-Pandemie

Das „Konzeptpapier Forschungspriorisierung – Konzept zur Priorisierung von Forschungsfragen und -themen in einer Pandemie“ richtet sich potenziell an alle Wissenschaftler:innen sowie Entscheidungsträger:innen auf übergeordneter Ebene (z. B. Politiker:innen, Einrichtungen von Forschungsförderung), die über die Ausrichtung von Forschungsinhalten im Gesundheitssektor entscheiden. Das Konzept wurde durch die Einbettung in das NUM im pandemischen Kontext entwickelt. Das NUM setzt in der Forschung auf Kooperation der Universitätskliniken statt auf Wettbewerb, sodass dessen Forschungsprojekte und Infrastrukturen einen hohen Grad an Interdisziplinarität und Weiterentwicklungsmöglichkeiten aufweisen. Aus diesem Grund handelt es sich um ein Produkt, welches zwar einen strukturierenden Rahmen für die Priorisierung von Forschungsfragen und -themen bietet, jedoch notwendige Adaptionen zulässt. In diesem Sinne ist das Konzept auch auf andere gesundheitliche Krisensituationen anwendbar und nicht auf den Einsatz in Pandemien beschränkt. Künftig soll es eine Ressource innerhalb der NUM-Infrastruktur darstellen, um ein agiles Pandemiemanagement zu gewährleisten.

Obwohl bereits mehrere internationale Ansätze zur Forschungspriorisierung vorliegen, sah man sich in der COVID-19-Pandemie mit der „Gleichzeitigkeit“ vieler grundlegender Herausforderungen konfrontiert:

1. Es galt, möglichst *schnell* sowohl den Erreger als auch dessen Ausbreitung und gesundheitliche Auswirkungen zu verstehen.
2. Zwar gab es bestehende Forschung zu Coronaviren und damit sogenannte indirekte Evidenz, aber Therapiemöglichkeiten für eine COVID-19-Erkrankung oder Maßnahmen zum Infektionsschutz mussten passgenau entwickelt und erforscht werden. Dazu wurden u. a. *neue Forschungsinfrastrukturen und Projekte geschaffen*, um die Krise zu bewältigen (vgl. NUM).
3. In einer akuten Krise besteht weniger zeitlicher Spielraum für *Versuch und Irrtum sowie für Fehler*, weshalb es von großer Bedeutung war, Forschungsvorhaben in zielführende Richtungen zu lenken.
4. Weiterhin *veränderten sich der* *Wissens- und Forschungsstand* rasant und die Anzahl an wissenschaftlichen Publikationen „explodierte“ geradezu [8]. Neue Forschungsfragen oder Folgefragen, die sich aus bisherigen Erkenntnissen ergaben, führen letztlich auch zur Notwendigkeit einer neuen Bewertung der Relevanz und ggf. zur neuen Ausrichtung von Forschung.

All diese Faktoren und die Tatsache, dass eine globale Krise wie eine Pandemie alle Lebensbereiche betrifft, macht den Pool an Forschungsfragen und -themen potenziell unendlich groß. Was das Konzept zur Forschungspriorisierung also leisten soll, ist eine Handreichung zur Etablierung von Strukturen der Forschungspriorisierung und zum Treffen von Entscheidungen darüber, welche Forschungsfragen aktuell relevant oder relevanter als andere sind und welche Dringlichkeit jeweils hinter einer Frage steht.

## Methodik: Von „CEOsys“ zu „PREPARED“

Die Priorisierung von Forschungsfragen in CEOsys basierte auf einem *Ranking* identifizierter Forschungsfragen, welches von Personen unterschiedlicher Fachdisziplinen und nach der Festlegung *einheitlicher Bewertungskriterien* durchgeführt wurde.

Die Bewertungskriterien sowie die Auswertungsmethode (Berechnung eines Rankings) orientierten sich am „Up-Priority Tool“ zur Aktualisierung klinischer Leitlinien der „Guidelines International Network (G-I-N) Updating Guidelines Working Group“ [2] und der damals aktuellsten Übersichtsarbeit zu Priorisierungsansätzen bei der Entwicklung gesundheitlicher Handlungsempfehlungen [9]. Diese Ansätze wurden durch eine orientierende Recherche bei international renommierten Forschungsorganisationen identifiziert. Der gesamte Prozess wurde an die Projektstrukturen und den pandemischen Kontext angepasst. Dazu zählten z. B. die Auswahl der priorisierenden Expert:innen aus verschiedenen Fachdisziplinen sowie die Notwendigkeit, den Vorgang unter Zeitdruck durchzuführen. Anhand der festgelegten Kriterien bewertete jede ausgewählte Person anonym und individuell jede Frage. Diese unabhängigen Einzelbewertungen wurden zu einem Mittelwert zusammengeführt, der den Rang einer Forschungsfrage im Gesamtranking ergab. So sollte eine möglichst nachvollziehbare, transparente und objektive Priorisierung erreicht werden. An diesem grundsätzlichen Prozess wurde bei der Weiterentwicklung festgehalten.

In der Projektlaufzeit von CEOsys wurde klar, wie wichtig eine gute Organisation von Priorisierungsprozessen für die Gesundheitsforschung ist, gerade im Hinblick auf weitere Gesundheitskrisen. In akuten Situationen kann es zu Personalausfall oder zu konkurrierenden Verpflichtungen von Priorisierungsverantwortlichen kommen. Solchen Hindernissen sollte konzeptionell entgegengewirkt werden. Die gesammelten Erfahrungen und Lessons Learned der Projektbeteiligten in CEOsys, Diskussionen mit wissenschaftlich Tätigen verschiedener Bereiche innerhalb von PREPARED (z. B. Versorgungsforschung, evidenzbasierte Medizin, Public Health) sowie eine weiterführende Literaturrecherche führten zu einem Konzept, das sowohl organisatorische als auch prozedurale Aspekte von Forschungspriorisierung einbezieht.

Da Forschungspriorisierung in hohem Maße kontextabhängig ist, kann es kein „Rezept“ geben, das standardmäßig alle Details eines Priorisierungsprozesses festlegt. So können z. B. die inhaltlichen Bewertungskriterien je nach Ziel der Priorisierung oder die priorisierenden Personen entsprechend benötigter Fachexpertise variieren.

## Inhalte des Konzeptpapiers Forschungspriorisierung

**Abb. 1:**
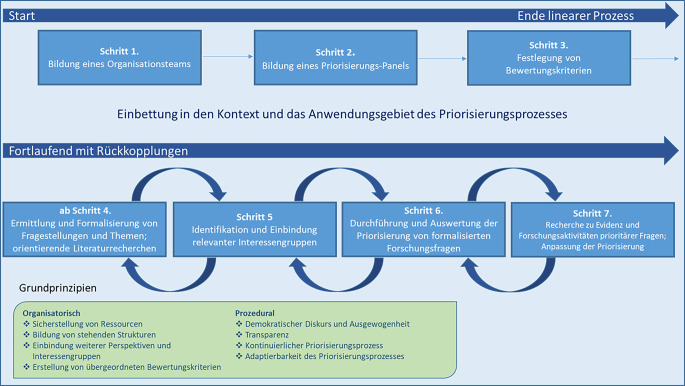
Prozess der Forschungspriorisierung für das Projekt „PREPARED“. Hinweise: *Gebogene Pfeile* sollen Rückkopplungsprozesse und damit eventuelle Wiederholungen der Schritte ab Schritt 4 darstellen. Grundprinzipien in der grünen Umrahmung gelten für den gesamten Prozess (Quelle: eigene Grafik aus „Konzeptpapier Forschungspriorisierung“ im Onlinematerial)

In dem adaptierbaren Konzept werden zunächst organisatorische und prozedurale Grundprinzipien für einen gelingenden Priorisierungsprozess aufgeführt. Bei der Umsetzung des vorgeschlagenen 7‑schrittigen Prozesses der Priorisierung sollten diese allgemeinen Prinzipien bestmöglich eingehalten werden (Abb. 1).

### Grundprinzipien bei der Priorisierung von Forschungsfragen und -themen

Als relevante *organisatorische Grundprinzipien* wurden folgende Aspekte identifiziert:

1. *Sicherstellung von Ressourcen für Priorisierungsprozesse:* Es ist sicherzustellen, dass Personen mit geeigneter methodischer und fachlicher Expertise sowie ausreichendem Zeitkontingent zur Verfügung stehen und finanziert werden.
2. *Bildung von stehenden Strukturen:* explizite Benennung und Etablierung von administrativen und inhaltlichen Verantwortlichkeiten.
3. *Einbindung weiterer Perspektiven und Interessengruppen:* Da nicht davon auszugehen ist, dass jegliche Expertise für eine informierte Priorisierung intern (z. B. im Forschungsverbund) vorhanden ist, sollten grundsätzlich wichtige externe Personengruppen bzw. Stakeholder identifiziert und eingebunden werden.
4. *Erstellung von übergeordneten Bewertungskriterien:* Zur Einheitlichkeit und Nachvollziehbarkeit sollten kontextspezifisch möglichst etablierte, breit akzeptierte Kriterien festgelegt werden, die von allen Beteiligten angewandt werden.

Als relevante *prozedurale Grundprinzipien* wurden folgende Aspekte identifiziert:

1. *Demokratischer Diskurs und Ausgewogenheit:* zielt auf eine möglichst hohe Neutralität im Priorisierungsprozess ab. Eine Gleichstellung der Mitglieder in Bezug auf Fachdisziplin und Hierarchie wird angestrebt.
2. *Transparenz:* Potenzielle Interessenkonflikte aller Priorisierenden sollten erfasst, offengelegt und im Priorisierungsprozess berücksichtigt werden. Ebenso sollten die Ergebnisse der Priorisierung transparent dargestellt werden.
3. *Kontinuierlicher Prozess:* Es ist davon auszugehen, dass eine Priorisierung regelmäßig aktualisiert oder mehrfach durchgeführt werden muss, da der Bedarf an Forschung variiert.
4. *Adaptierbarkeit des Priorisierungsprozesses:* Sowohl der vorgeschlagene 7‑schrittige Prozess als auch dessen Umsetzung im konkreten Anwendungsfall sollten als abwandelbar begriffen werden. Änderungen sollten sichtbar dokumentiert, begründet und nicht zulasten der übrigen Grundprinzipien vorgenommen werden.

### 7-schrittiger Prozess der Priorisierung von Forschungsfragen und -themen

Die 7 Schritte sind unterteilt in einen linearen Prozess (Schritte 1–3; vorbereitend) und einen Prozess mit Rückkopplungen (Schritte 4–7; durchführend; Abb. 1). Mit dieser Unterteilung soll erreicht werden, dass in der Präpriorisierungsphase alle notwendigen Überlegungen getroffen werden, um eine systematische Priorisierung im Anschluss effizient zu ermöglichen. Die Schritte werden zunächst kurz beschrieben und die jeweiligen Kernaufgaben in Tab. 1 übersichtlich zusammengefasst.

**Tab. 1:** Übersicht der Schritte 1–7 des Prozesses zur Forschungspriorisierung im Projekt „PREPARED“

Schritt	Ziel	Verantwortliche Personen	Aktivitäten und weitere relevante Elemente
1. Bildung eines Organisationsteams	Fortlaufende Koordination und fortlaufendes Monitoring	Z. B. Projektkoordinator:innen, Expert:innengremium	-Bildung des Organisationsteams und Anbindung an Strukturen des Anwendungskontextes -Möglichst an einem Standort tätige Mitglieder mit Kenntnissen in Projektkoordination/Projektmanagement -Enge Vernetzung mit Priorisierungs-Panel (Schritt 2)
2. Bildung eines Priorisierungs-Panels	Festlegung der Verantwortlichen zur Durchführung der Forschungspriorisierung	Organisationsteam unter Einbezug weiterer Expert:innen und/oder verantwortliche Personen innerhalb des Priorisierungskontextes (z. B. Mitglieder eines Forschungsprojekts)	-Bildung/Benennung des Priorisierungs-Panels -Anbindung an Struktur des Anwendungsgebiets; über Standorte verteilt (Risikostreuung bei kurzfristigem Ausfall) -Festlegung einer Panel-Leitung mit festgelegter Rangfolge bei Ausfall -Risikoanalyse zu konkurrierenden Verpflichtungen der Panel-Mitglieder -Sicherstellung einer Doppelbesetzung für jede Fachdisziplin und für Mitglieder mit erhöhtem Risiko für konkurrierende Verpflichtungen -Ausfüllen eines Letter-of-Intent+Declaration-of-Interest-Formulars durch jedes Panel-Mitglied
3. Festlegung von Bewertungskriterien	Erstellen des Kriterienkatalogs, inklusive Anleitung oder Beschreibung zur Anwendung	Organisationsteam und Priorisierungs-Panel + ggf. weitere Personen (z. B. zur Recherche)	-Recherche nach passenden Kriterien für die Priorisierung -Überprüfung der Kriterien auf notwendige Anpassungen, ggf. Hinzunahme oder Aussparung von Kriterien -Gemeinsame Entscheidung über den Eingang von Kriterien zur Priorisierung -Pilotierung der Kriterien in einer beispielhaften Priorisierung -Erstellung von Erläuterungen und Fallbeispielen zu den einzelnen Kriterien
4. Ermittlung und Formalisierung von Fragestellungen und Themen	Sammlung und Formalisierung von zu priorisierenden Forschungsfragen und -themen, inklusive Festlegung der Zuständigkeiten; Durchführung von orientierenden Literaturrecherchen	Einreichende Personen oder Institutionen und/oder Organisationsteam und/oder Priorisierungs-Panel + ggf. Einbezug weiterer wissenschaftlicher Mitarbeiter:innen	-Festlegung von Wegen und Zuständigkeiten für unten genannte Aktivitäten -Erstellung und Nutzung von Templates zur Einreichung von Fragestellungen -Organisationsteam verwaltet Fragestellungen und Themen und fasst Dopplungen zusammen -Ggf. Vorselektion von Fragestellungen -Formalisierung/Operationalisierung der Fragen: Berücksichtigung von Prinzipien der Erstellung von Forschungsfragen, z. B. Nutzung von Frameworks für Forschungsfragen (z. B. PICO: Population, Intervention, Comparator, Outcomes) -Ggf. schnelle, orientierende Literaturrecherchen zu den operationalisierten Forschungsfragen -Fragestellungen werden nur in Ausnahmefällen vom Panel selbst eingebracht
5. Identifikation und Einbindung relevanter Interessengruppen	Einbindung externer, möglichst repräsentativer Interessengruppen zur Berücksichtigung relevanter Perspektiven	Priorisierungs-Panel, unterstützt durch Organisationsteam und Vertreter:innen ausgewählter Interessengruppen	-Identifikation von weiteren benötigten Perspektiven -Ableitung einzubindender Interessengruppen und Versendung von Anfragen -Bestimmung der Rolle hinzugezogener Interessengruppen -Ggf. Ergänzung weiterer relevanter Fragestellungen -Letter of Intent für externe Beteiligte -Conflict-of-Interest-Formular für externe Beteiligte
6. Durchführung und Auswertung der Priorisierung von formalisierten Forschungsfragen	Festlegung der prioritären Forschungsfragen und deren Rangfolge	Priorisierungs-Panel + ggf. weitere extern eingebundene Interessengruppen und Organisationsteam (formelle Organisation)	-Einschätzung der Priorität für jede Forschungsfrage basierend auf den festgelegten Bewertungskriterien -Erstellung einer oder mehrerer Ranglisten von Forschungsfragen -Offene Felder für Begründungen und Ausführungen abseits der festgelegten Kriterien -Dokumentation des Ratings *Einige mögliche Darstellungsoptionen der Priorisierung:* -Eintrag der Fragestellungen in eine Priorisierungsmatrix -Getrennte Ranglisten für verschiedene Themenbereiche oder Fachdisziplinen -Gesamtrangliste mit verschiedenen Disziplinen -Getrennte Ranglisten nach anzuwendender Forschungsmethodik, sofern diese schon bestimmt wird (Modellierungen, Evidenzsynthesen, klinische Studie etc.) -Template (z. B. Dokument, Survey), das Forschungsfragen und Bewertungskriterien beinhaltet
7. Recherche zu Evidenz und Forschungsaktivitäten prioritärer Fragen	Erfassung des Forschungsstands zu priorisierten Forschungsfragen und ggf. Anpassung der Rangfolge	Eingebundene wissenschaftliche Mitarbeitende (z. B. Expert:innen für Literaturrecherche) und/oder Organisationsteam und/oder Priorisierungs-Panel und/oder mit dem Panel assoziierte und extern beauftragte Methodiker:innen	-Vorrecherche und Sichtung der Literatur zu Forschung, weiteren aktuellen (und geplanten) Forschungsaktivitäten sowie zu politischem und gesellschaftlichem Diskurs der priorisierten Forschungsfragen -Dokumentation der Rechercheergebnisse mit Angabe von genutzten Datenbanken und Quellen. Evtl. Anpassung der Priorisierungsergebnisse: Reduktion der Anzahl von Fragen, stärkere Spezifizierung oder Veränderung der Rangfolge von Fragen

#### Schritt 1 – Bildung eines Organisationsteams

Gerade in größeren Forschungsprojekten oder in Organisationen, in denen Forschung priorisiert werden muss ist es hilfreich ein administrativ arbeitendes Team zu etablieren. Dieses Team ist dafür verantwortlich alle notwendigen Schritte und Abläufe im Blick zu behalten, Termine zu planen oder Interessenkonflikte zu erheben. Die Zuständigkeiten können im Anwendungsfall ggf. ergänzt werden. Wichtig ist, dass die Aufgaben dieses Organisationsteams nicht in der eigentlichen Priorisierung liegen, sondern diese administrativ unterstützen. In einem kleinen Projekt oder Team kann dies ggf. auch von einer Person übernommen und auf ein formales Gremium verzichtet werden, wenn sich der administrative Aufwand in Grenzen hält. Eine hervorzuhebende Aufgabe ist die Organisation des zu etablierenden Priorisierungs-Panels (s. Schritt 2) und die Auswahl der Mitglieder anhand fachlicher Expertise (sofern diese nicht ohnehin bereits feststehen). Eine weitere zentrale Aufgabe ist die Bündelung eingehender, zu priorisierender Fragestellungen und -themen.

#### Schritt 2 – Bildung eines Priorisierungs-Panels

Schritt 2 sieht die Auswahl von Personen vor, die die Priorisierung durchführen, d. h. die Forschungsfragen und -themen hinsichtlich ihrer Relevanz bewerten. Das entstehende Gremium nennt sich hier *Priorisierungs-Panel*. Es sollte interdisziplinär ausgerichtet sein bzw. die notwendige fachliche und/oder methodische Expertise abdecken. Da im Projekt CEOsys eine Priorisierung für durchzuführende Evidenzsynthesen im Fokus stand, waren hier z. B. Expert:innen der evidenzbasierten Medizin unabdingbar. Grundsätzlich sollten auch Vertreter:innen pro Mitglied benannt werden, um die Arbeitsfähigkeit des Panels auch bei Doppelverpflichtungen oder im Krankheitsfall sicherzustellen. Die Zusammenstellung und auch die Größe eines solchen Panels ist stark vom Kontext der Priorisierung abhängig, sollte aus pragmatischen Gründen aber dennoch nicht zu groß sein und bei Bedarf durch weitere Interessenvertretungen (siehe Schritt 5) inhaltlich beraten werden.

#### Schritt 3 – Festlegung von Bewertungskriterien

Die Kriterien für die Priorisierung sind das Herzstück des Prozesses und ihre Festlegung erfordert Zeit für die Recherche, Auswahl und Formulierung sowie einen breiten Konsens. Auch die Kriterien richten sich nach dem Priorisierungskontext. Geht es im Rahmen der Priorisierung z. B. um wissenschaftliche Politikberatung, so wäre das Kriterium *politische Relevanz* naheliegend. Außerdem sollte eine digitale Vorlage erstellt werden, in der die zu priorisierenden Forschungsfragen bewertet werden können (ausfüllbares Format). Die folgenden Kriterien sind Beispiele für die Bewertung, ob eine Forschungsfrage prioritär zu bearbeiten ist:

- individuelle und bevölkerungsbezogene Krankheitslast,
- Patient:innensicherheit,
- Gesundheit von Mitarbeitenden im Gesundheitssektor,
- Adressieren einer Forschungslücke,
- Kosten-Nutzen-Bewertung,
- Machbarkeit,
- politische Relevanz.

Ein Bespiel für eine vollständige Bewertungsmatrix findet sich im Anhang und entstammt dem Prozess aus CEOsys (s. Onlinematerial, S. 32). In der Literatur finden sich bereits mögliche Kriterien zur Bewertung, an denen sich Gruppen orientieren können [10].

#### Schritt 4 – Ermittlung und Formalisierung von Fragestellungen und Themen

Ab diesem Schritt werden die in Schritt 1–3 festgelegten Organisationsstrukturen benötigt, um eine kontinuierliche Priorisierung umzusetzen. In seltenen Fällen wird es zu genau einem Zeitpunkt genau einen Pool an Fragestellungen geben, die dann abschließend priorisiert werden können. Wahrscheinlicher ist es, dass sich im Verlauf weitere Fragen ergeben, bei denen jeweils entschieden werden muss, ob sie formal priorisiert werden. Es sollte unbedingt geklärt werden, welche Personengruppen zu welchen Zeitpunkten berechtigt sind, Fragen und Themen einzubringen und wie diese das Priorisierungs-Panel erreichen. Wichtig ist hier, eingegangene Fragestellungen oder allgemein formulierte Themen zunächst in konkrete Forschungsfragen zu übersetzen. Sie sollten so formuliert werden, dass sie mit wissenschaftlichen Methoden untersucht werden können. Man kann nicht davon ausgehen, dass alle eingehenden Fragen direkt die notwendige Klarheit und Präzision aufweisen, die für Forschungsaktivitäten unabdinglich sind. Auch dieser Schritt kann Zeit in Anspruch nehmen, da für eine möglichst zielführende Priorisierung und Forschung präzise formulierte Forschungsfragen erforderlich sind. Sie sind in der Regel der Ausgangspunkt eines Forschungsvorhabens und an ihnen richten sich die Machbarkeit und die Methodik aus. An diesem Punkt im Priorisierungsprozess können bereits orientierende Literaturrecherchen zu eingegangenen Fragestellungen notwendig sein.

#### Schritt 5 – Identifikation und Einbindung relevanter Interessengruppen

Auch interdisziplinäre Priorisierungs-Panels können höchstwahrscheinlich nicht alle Perspektiven abdecken, die ausschlaggebend für eine Bewertung der Priorität sind. Wichtige Informationen und Eindrücke aus Versorgung, Forschung oder Politik können diese Bewertung verändern. Einzubindende Interessengruppen richten sich nach den jeweiligen Themen und umfassen meist entweder Verantwortungs- oder Entscheidungsträger:innen (z. B. Gesundheitspersonal) oder vom Thema betroffene Personen (z. B. Patientenvertretungen). Es sollten gemeinsame Sitzungen des Priorisierungs-Panels mit den identifizierten Vertreter:innen von Interessengruppen erfolgen. Da es die Machbarkeit übersteigen würde, spezifische Interessenvertreter:innen pro Forschungsfrage zu akquirieren, sollte die Auswahl übergeordneter erfolgen. Je nach Gebiet gibt es Institutionen, die bei der Identifizierung behilflich sein können. Ein Beispiel wäre die Arbeitsgemeinschaft der Wissenschaftlichen Medizinischen Fachgesellschaften (AWMF), die den Zugang zu relevanten Fachgesellschaften bahnen kann.

#### Schritt 6 – Durchführung und Auswertung der Priorisierung von formalisierten Forschungsfragen

Jedes Panel-Mitglied sollte die formalisierten Forschungsfragen nun anhand der in Schritt 3 festgelegten Kriterien einzeln und anonym mittels des vorbereiteten Formulars bewerten (verblindetes Verfahren). Sollten nur Fragen eines einzigen Fachbereichs priorisiert werden, ist zu überlegen, ob nur der entsprechende Teil des Panels einbezogen wird. Die Einzelergebnisse werden vom Organisationsteam quantitativ und, falls notwendig, auch qualitativ ausgewertet. Die Art der Auswertung sollte vorab festgelegt werden und sich am Formular zur Bewertung orientieren (z. B. Skalierung 1–7 der Antwortmöglichkeiten, Umgang mit Anmerkungen, Mittelwertbildung, mögl. Gewichtung von Kriterien). Nach und vor dieser formalen Bewertung sind Austausch und Diskussionen des Priorisierungs-Panels in geplanten Sitzungen sinnvoll, die zur Klärung von Unsicherheiten beitragen können. Die Ergebnisse der Panel-Bewertung sollten unter Einbindung der relevanten Interessengruppen interpretiert werden. Am Ende von Schritt 6 sollen eine Bewertung der Relevanz und Dringlichkeit sowie eine daraus resultierende Rangfolge der Forschungsfragen vorliegen.

#### Schritt 7 – Recherche zu Evidenz und Forschungsaktivitäten prioritärer Fragen und Anpassung der Priorisierung

Schon vor der formalen Priorisierung sollten orientierende Recherchen zum aktuellen Forschungs- und Bearbeitungsstand zu jeder Forschungsfrage erfolgt sein, um informierte Entscheidungen über die Priorität zu ermöglichen. Da die Mitglieder eines Priorisierung-Panels aufgrund ihrer fachlichen Expertise ausgewählt wurden, sollte der themenbezogene Forschungsstand aber grundsätzlich bekannt sein. Recherchen sollten in jedem Fall vor Abschluss der Priorisierung und vor der Initiierung von Forschungsprojekten erfolgen. Aufgrund aktueller Ereignisse, Entwicklungen und geplanter Forschungsvorhaben ist hierbei eine zeitliche Nähe der Recherchen zur Bewertung in Schritt 6 anzustreben. Die Ergebnisse dieser Recherchen können potenziell neue bzw. andere Fragen aufwerfen oder die bisherige Rangfolge infrage stellen.

Generell ist davon auszugehen, dass Priorisierung *kein* linearer, statischer Prozess ist und dass Priorisierungsrunden im Verlauf mehrfach durchgeführt oder Rangfolgen angepasst werden müssen. Nichtsdestotrotz sollten auch Entscheidungen über (vorläufig) geltende Priorisierungen klar und transparent kommuniziert werden, damit geeignete Forschung initiiert werden kann und Entscheidungen für Außenstehende (z. B. Allgemeinbevölkerung) nachvollziehbar sind.

## Forschungspriorisierung in der Zukunft

Gesundheitskrisen werden weiterhin Teil unserer Realität im Gesundheitswesen sein. Wann erneut eine Pandemie ausbricht, ist ungewiss, aber Probleme wie Antibiotikaresistenzen, Fachkräftemangel im Gesundheitswesen oder steigende Gesundheitskosten beschäftigen die Forschung, Politik und Gesundheitsversorgung bereits intensiv. Letztlich müssen Entscheidungen darüber fallen, welche Forschung notwendig ist, um unser Gesundheitssystem und die medizinische Versorgung krisenfest zu gestalten, wie man sich auf solche Krisen vorbereitet und auch wie man eine adäquate Aufarbeitung erreicht. Die Notwendigkeit von Priorisierung kann außerdem auch auf den sonstigen (nicht krisenbezogenen) Forschungsbetrieb übertragen werden, da Ressourcenverteilung stetig erfolgen muss. Der Unterschied besteht in der höheren benötigten *Reaktionsgeschwindigkeit* von Gesundheitssystem und -personal, von Politik und Forschung.

Generell plädieren wir dafür, eine strukturierte Priorisierung von Forschungsfragen und -themen stärker als Grundsatz in der deutschen Forschungslandschaft zu verankern, um bedarfsgerechte, transparente Forschung zu betreiben. Die Entscheidungen darüber, welchen Themen Relevanz beigemessen wird, sollten nach transparenten, systematischen Prozessen und anhand nachvollziehbarer Kriterien getroffen werden. Dabei sollten relevante Interessengruppen einbezogen, auf bereits vorhandenes Wissen aufgebaut und aktuelle Entwicklungen aufgegriffen werden.

Das hier vorgestellte Konzeptpapier bietet einen aus der COVID-19-Pandemie hervorgegangenen Ansatz zur Priorisierung von Forschungsfragen und kann als Entscheidungshilfe für die Initiierung von Forschungsprojekten anhand der bewerteten Relevanz und Dringlichkeit von Forschungsfragen genutzt werden. Das Konzept knüpft dabei an einen bereits angewandten Prozess im Bereich der Priorisierung von Forschungsfragen zu Evidenzsynthesen und Leitlinienprojekten an, verfolgt aber eine Ausrichtung mit breitem potenziellen Anwendungsgebiet. Bisher hat noch keine Validierung des Prozesses in seiner aktuellen Form stattgefunden. Weiterentwicklungen sind daher möglich und wahrscheinlich. Der Prozess sollte in verschiedenen Kontexten und unter wissenschaftlicher Begleitung (Evaluation) angewandt werden. Er soll in Zukunft noch detailliertere Anweisungen und weitere Anwendungsbeispiele beinhalten und mögliche Herausforderungen in der Umsetzung berücksichtigen. Eine solche Herausforderung könnten z. B. konkurrierende Bewertungskriterien darstellen, wenn etwa die *individuelle Krankheitslast* hoch und die *bevölkerungsbezogene Krankheitslast* niedrig eingestuft wird. Um der zeitlichen Dringlichkeit in Krisen Rechnung zu tragen, sollte die infrastrukturelle Etablierung von Priorisierungsansätzen außerdem in Zeiten stattfinden, in denen die Kapazitäten dafür bestehen. Deshalb sollten insbesondere die Aktivitäten der linearen Schritte 1–3 so weit wie möglich vorbereitet werden. Zum einen, um im Krisenfall tatsächlich so schnell und koordiniert wie möglich agieren zu können, und zum anderen, um weiteren auftretenden Herausforderungen entgegenzuwirken und mögliche Anpassungen am Prozess vorzunehmen. Es wäre eine vertane Chance, den in der Pandemie sichtbar gewordenen Bedarf an klaren Strukturen, Prozessen und Zuständigkeiten zur Forschungspriorisierung nicht ausreichend zu adressieren.

Über eine Erprobung des hier vorgeschlagenen Konzeptes hinaus sollte auch eine umfassende Aufbereitung internationaler Ansätze zur Forschungspriorisierung durchgeführt werden. Zwar beinhaltet die Entwicklung dieses Konzepts eine orientierende Literaturrecherche, jedoch könnten durch eine systematische Recherche und Analyse internationaler Priorisierungskonzepte weitere Aspekte einbezogen werden. Insbesondere Implikationen aus der Pandemiezeit könnten für weitere Gesundheitskrisen im Umgang mit Priorisierung und Ressourcenallokation wegweisend sein.

## Supplementary Information

Konzeptpapier Forschungspriorisierung

## Footnotes

1 Netzwerk Universitätsmedizin: www.netzwerk-universitaetsmedizin.de/. Letzter Zugriff am 13.03.2024.

2 COVID-19-Evidenz-Oekosystem*:* www.covid-evidenz.de/. Letzter Zugriff am 13.03.2024.

3 PREparedness and PAndemic REsponse in Deutschland: www.num-prepared.de/. Letzter Zugriff am 13.03.2024.
