# Supplementary material for: Prioritization of research questions in health crises—presentation of a concept developed during the COVID-19 pandemic
Source: Bundesgesundheitsblatt Gesundheitsforschung Gesundheitsschutz. 2024 Dec 5;68(1):97–104. [Article in German] doi: 10.1007/s00103-024-03985-4 (PMC11732861; doi:10.1007/s00103-024-03985-4)
Supplement: Supplementary file 1 — Konzeptpapier Forschungspriorisierung [file 103_2024_3985_MOESM1_ESM.pdf]

Konzeptpapier Forschungspriorisierung

Konzept zur Priorisierung von Forschungsfragen und -themen in einer Pandemie

Arbeitspaket 1.3 Schnelle Forschungspriorisierung und -initiierung NUM 2.0 PREPARED

„PREparedness and PAndemic REsponse in Deutschland“

basierend auf Vorarbeiten von Arbeitspaket 1 – NUM 1.0 CEOsys

„COVID-19-Evidenz-Ökosystem“

Erstellt durch

Annika Ziegler[1], Angela M. Kunzler[1], Sebastian-Voigt-Radloff[1,2], Joerg J. Meerpohl[1,3] (Freiburg)

[1] Institut für Evidenz in der Medizin, Universitätsklinikum Freiburg, Medizinische Fakultät, Albert-Ludwigs-Universität Freiburg, Freiburg, Deutschland

[2] Institut für medizinische Biometrie und Statistik, Universitätsklinikum Freiburg, Medizinische Fakultät, Albert-Ludwigs-Universität Freiburg, Freiburg, Deutschland

[3] Cochrane Deutschland, Cochrane Deutschland Stiftung, Freiburg, Deutschland

Weitere Beteiligte des AP1.3

Christian Apfelbacher[4], Heike Heytens[4] (Magdeburg)

[4] Institut für Sozialmedizin und Gesundheitssystemforschung, Medizinische Fakultät, Universitätsklinikum Magdeburg, Magdeburg, Deutschland

Jochen Schmitt[5] (Dresden)

[5] Zentrum für evidenzbasierte Gesundheitsversorgung, Universitätsklinikum Dresden, Dresden, Deutschland

Onnen Mörer[6], Simone Scheithauer[7] (Göttingen)

[6] Klinik für Anästhesiologie und Intensivmedizin, Universitätsklinikum Göttingen, Göttingen, Deutschland

[7] Institut für Krankenhaushygiene und Infektiologie, Universitätsklinikum Göttingen, Göttingen, Deutschland

Thomas Kaiser[8] (Köln)

[8] Institut für Qualität und Wirtschaftlichkeit im Gesundheitswesen (IQWiG), Köln, Deutschland

Katrin Balzer[9] (Lübeck)

[9] Sektion für Forschung und Lehre in der Pflege, Universitätsklinikum Schleswig-Holstein, Lübeck, Deutschland

Anna Levke Bruett[10] (Oldenburg)

[10] Department für Versorgungsforschung, Fakultät Medizin und Gesundheitswissenschaften, Carl von Ossietzky Universität Oldenburg, Oldenburg, Deutschland

Monika Klinkhammer-Schalke[11] (Regensburg)

[11] Tumorzentrum Regensburg, Zentrum für Qualitätssicherung und Versorgungsforschung, Fakultät für Medizin der Universität Regensburg, Regensburg, Deutschland

Acknowledgement

Weitere Beteiligte innerhalb der Vorarbeiten aus CEOsys (AP1)

Nico T. Mutters[12] (Bonn)

[12] Institut für Hygiene und Öffentliche Gesundheit, Universitätsklinikum Bonn, Bonn, Deutschland

Winfried Kern[13] (Freiburg)

[13] Abteilung Infektiologie, Klinik für Innere Medizin II, Universitätsklinikum Freiburg, Medizinische Fakultät, Albert-Ludwigs-Universität Freiburg, Freiburg, Deutschland

Martin Golinski[6], Daniel Heise[6], Nils Kunze-Szikszay[6] (Göttingen)

[6] Klinik für Anästhesiologie und Intensivmedizin, Universitätsklinikum Göttingen, Göttingen, Deutschland

Caroline Hirsch[14], Nicole Skoetz[14] (Köln)

[14] Evidenz-basierte Medizin, Klinik I für Innere Medizin, Medizinische Fakultät und Uniklinik Köln, Universität zu Köln, Köln, Deutschland

Lisa Pfadenhauer[15] (München)

[15] Institut für med. Informationsverarbeitung Biometrie und Epidemiologie, Medizinische Fakultät, Ludwig-Maximilians-Universität München, München, Deutschland

Inhalt

1. Hintergrund – Warum wir Forschungsfragen und -themen priorisieren müssen ... 6

2. Ziel des Konzeptpapiers ... 6

3. Methodik ... 7

4. Grundprinzipien für die Priorisierung von Forschungsfragen und -themen im Kontext einer Pandemie ... 8

4.1 Organisatorische Grundprinzipien ... 8

4.2 Prozedurale Grundprinzipien ... 9

5. Schritte der Priorisierung von Forschungsfragen und -themen im Kontext einer Pandemie ... 11

Schritt 1. Bildung eines Organisationsteams ... 13

Schritt 2. Bildung eines Priorisierungs-Panels ... 14

Schritt 3. Festlegung eines Katalogs von Bewertungskriterien ... 16

Schritt 4. Ermittlung von Fragestellungen und Themen zur Priorisierung und Formalisierung von Forschungsfragen ... 18

Schritt 5. Frühzeitige Identifikation und Einbindung relevanter Perspektiven und Interessengruppen ... 20

Schritt 6. Durchführung und Auswertung der Priorisierung von formalisierten Forschungsfragen ... 22

Schritt 7. Recherche zu Evidenz und Forschungsaktivitäten zu den jeweiligen Fragestellungen und Anpassung der Priorisierungsergebnisse ... 24

Regelmäßige Wiederholung der Schritte 4-7 im dynamischen Pandemiegeschehen ... 25

6. Ausblick ... 26

6.1 Einschätzung zum zeitlichen Ablauf des Priorisierungsprozesses ... 26

6.2 Schritte nach der Priorisierung von Forschungsfragen ... 26

7. Weitere Aspekte der Forschungspriorisierung im Kontext einer Pandemie ... 27

7.1 Fragenkatalog relevanter wissenschaftlicher Fragestellungen aus der COVID-19-Pandemie (Priorisierungsmatrix) ... 27

7.2 Etablierung einer repräsentativen Bürger-Kohorte in Deutschland ... 28

7.3 Berücksichtigung von Ergebnissen aus Modellierungen (Value of Information Analysis) ... 29

8. Weitere Ressourcen ... 31

9. Literatur 31

10. Anhang 32

1. Hintergrund – Warum wir Forschungsfragen und -themen priorisieren müssen

Globale Krisen wie die COVID-19-Pandemie stellen gesundheitlich, wirtschaftlich und sozial große, disruptive Ereignisse dar. In allen Altersgruppen und in allen Settings können sie die öffentliche Gesundheit sowie die klinische Versorgung bedrohen und erfordern – besonders in der Initialphase – ein rasches, zeitkritisches Handeln.

Während der COVID-19-Pandemie waren Entscheidungsträger*innen in der Politik und im Gesundheitswesen sowie in weiteren Handlungsfeldern darauf angewiesen, auf wissenschaftliche Empfehlungen zur Diagnostik von SARS-CoV-2, zur Infektionsprävention und Behandlung von COVID-19, zum Pandemiemanagement, zu möglichen Auswirkungen der Erkrankung sowie pandemiebezogener Maßnahmen für verschiedene Bevölkerungsgruppen zurückgreifen zu können. Es bestand ein dringender Bedarf an kontextspezifischen, aktuellen, vertrauenswürdigen und adäquat aufgearbeiteten wissenschaftlichen Erkenntnissen, um evidenzgeleitete Entscheidungen zu ermöglichen.

Die hohe Dynamik in einer Pandemie erfordert eine kontinuierliche Anpassung der Relevanz und Dringlichkeit von Forschungsfragen und -themen, da diese möglichst den gesundheitspolitischen und gesellschaftlichen Bedarf adressieren sollen. Ressourcen wie Zeit, Personal und finanzielle Mittel sind oft begrenzt, während gleichzeitig die Anzahl von wissenschaftlichen Publikationen und auch von Fragestellungen im Pandemieverlauf rapide zunimmt.

Auf der anderen Seite gibt es zu Beginn oder für akut aufkommende Aspekte einer Pandemie bisweilen noch keine oder limitierte Evidenz, sodass Forschung erst initiiert werden muss. Vor diesem Hintergrund müssen vorhandene Ressourcen und Kapazitäten aus verschiedenen gesundheitsbezogenen Disziplinen (z. B. Diagnostik, ambulante / stationäre Versorgung, Intensiv- und Palliativversorgung, Hygiene und Infektionsprävention, Public Health, Psychologie und Psychiatrie) sorgfältig eingesetzt werden.

Eine praktikable Methodik zur Priorisierung von Forschungsfragen und -themen ist daher unverzichtbar. Sie gewährleistet den transparenten, bedarfsgerechten Einsatz von Ressourcen in der Forschung. Beispiele in einer Pandemie können auf gesundheitspolitischer Ebene die Priorisierung von Forschung zu Maßnahmen des Infektionsschutzes oder im klinischen Bereich die Priorisierung von Forschungsfragen zum Erreger oder zu Behandlungsmaßnahmen sein.

2. Ziel des Konzeptpapiers

Das vorliegende Konzept kann herangezogen werden, um festzulegen, welche identifizierten Forschungsfragen oder -themen so relevant sind, dass sie gegenüber anderen für eine wissenschaftliche Bearbeitung priorisiert werden sollten. Die Unterscheidung in Fragen und Themen

wird vorgenommen, da nicht immer davon ausgegangen werden kann, dass bereits spezifische Fragestellungen im Sinne einer gut operationalisierten Forschungsfrage aufkommen.

Präsentiert wird ein strukturierter und transparenter Ansatz zur Priorisierung von wissenschaftlichen, gesundheitsbezogenen Fragestellungen und Themen, der zur Vorbereitung und Bewältigung von zukünftigen, potenziellen Pandemien und gesundheitlichen Krisen beitragen soll. Er beschreibt Grundprinzipien und einen schrittweisen Prozess. Ergebnis einer solchen Priorisierung ist eine Liste an prioritären Fragestellungen, die in einer kriteriengeleiteten formalen Bewertung ermittelt werden. So erhalten Forschende, politische Entscheidungsträger*innen und Förderinstitutionen passend zum aktuellen Pandemiegeschehen Klarheit über dringlich erforderliche Forschungsbereiche.

Kontext der Priorisierung

Der Kontext bestimmt und legitimiert die Ziele, zeitlichen Vorgaben und Adressaten der Priorisierungsergebnisse. So hat z.B. die Priorisierung von Forschungsthemen für gesundheitspolitische Entscheidungen zur Forschungsförderung oder zum Infektionsschutz der Bevölkerung andere Ziele, Zeitvorgaben und Adressaten als die Priorisierung von konkreten Forschungsfragen innerhalb einer bereits geförderten Verbundforschung zum Thema SARS-CoV-2.

Das hier dargestellte Priorisierungskonzept wurde im Rahmen des Pandemic Preparedness Projektes PREPARED für die Nutzung im Netzwerk Universitätsmedizin (NUM, 2. Förderphase) entwickelt und bezieht sich auf den breiten Kontext von Forschungsverbundprojekten in Pandemie-Situationen. Es kann jedoch für andere Kontexte adaptiert werden, z.B. für andere gesundheitliche Krisensituationen, die für die klinische Versorgung und öffentliche Gesundheit von (inter-)nationaler Tragweite sind. Diese Adaptationsmöglichkeiten finden sich auch im PREPARED-Gesamtkonzept wieder.

Das Priorisierungskonzept soll eine Ressource für verschiedene Bereiche der PREPARED-Infrastruktur darstellen, welche zukünftig eine tragende Rolle in der Vorbereitung auf und im Management von Pandemien und weiteren Gesundheitskrisen spielen soll. Zum genaueren Verständnis der Funktionalitäten innerhalb von PREPARED und seiner Ziele und Akteure wird auf das in der Projektlaufzeit (08/2023-12/2024) entstandene Arbeitspapier verwiesen (Entwurfsfassung: Implikationen für ein zukunftsfähiges PREPARED Konzept) .

3. Methodik

Das vorliegende Konzept basiert auf Vorarbeiten zur Priorisierungsmethodik, die im Projekt CEOsys („COVID-19 Evidenz-Ökosystem") entwickelt und genutzt wurden. CEOsys war ein Teilprojekt der ersten Förderphase des NUM. Die Methodik diente zur Priorisierung von Versorgungsfragen bzw. maßnahmenbezogenen Fragestellungen als Basis für die Evidenzsynthesen- und Leitlinienerstellung in den Bereichen „Klinische Patientenversorgung“ und „Public Health“. Ausgehend vom Priorisierungsprozess aus CEOsys berücksichtigt dieses Konzeptpapier die identifizierten Herausforderungen einer Forschungspriorisierung im Zuge einer hochdynamischen Pandemiesituation. Es bindet entsprechende „Lessons Learned“ ein und leitet ein adaptiertes

generisches Gesamtkonzept zur Priorisierung von Forschungsfragen im Pandemiekontext ab. Generisch meint hier eine Priorisierung, die nicht nur Fragen zu Evidenzsynthesen fokussiert, sondern darüber hinaus auch zur Themen- und Fragenauswahl in der Primärforschung und der Forschungsförderung herangezogen werden kann.

Aus dem NUM-2-Projekt PREPARED sind außerdem Erfahrungsberichte sowie klinisches und methodisches Expertenwissen verschiedener Fachdisziplinen eingeflossen. Zudem wurden Aspekte einer ergänzenden Literatursichtung zu Priorisierungsprozessen und derzeitigen methodischen Empfehlungen ausgewählter Institutionen (Cochrane Methods Priority Setting, James Lind Alliance, Weltgesundheitsorganisation) einbezogen. Diese sind unter „Weitere Ressourcen“ aufgeführt.

Weiterentwicklung des Konzepts

Das aktuelle Konzept soll im Rahmen nachfolgender NUM-Aktivitäten weiterentwickelt und evaluiert werden. Möglich ist dies innerhalb der in PREPARED geplanten Infrastruktur. Speziell sind hier die Kernfunktionalitäten „Evidenzsynthesen für vertrauenswürdige Empfehlungen“ (ESVE) und das organisatorische „Rapid Reaction and Response Cockpit“ (R3-Cockpit) zu nennen. Hier sollen Netzwerke zum Pandemie-Management mit dauerhaft eingebundenen Forscher*innen, Kliniker*innen und weiteren relevanten Interessengruppen gebildet werden. Zudem kann das Konzept auch als Leitfaden für weitere NUM-Projekte, insbesondere für NAPKON-TIP („Nationales Pandemie Kohorten Netz – Therapeutische Interventionsplattform“) und CollPan („Collateral Effects of Pandemics“) sowie zukünftige Priorisierungsvorhaben innerhalb des NUM genutzt werden.

4. Grundprinzipien für die Priorisierung von Forschungsfragen und -themen im Kontext einer Pandemie

Die Priorisierung von Forschungsfragen und -themen unterliegt acht organisatorischen und prozeduralen Grundprinzipien, die in PREPARED und CEOsys für verschiedene Priorisierungskontexte als zielführend erachtet wurden. Die Anwendung der Grundprinzipien wird in dem schrittweisen Priorisierungsprozess detaillierter beschrieben (s. Kapitel 5).

4.1 Organisatorische Grundprinzipien

Sicherstellung von Ressourcen für Priorisierungsprozesse

Entsprechende Ressourcen für ein Priorisierungsverfahren sollten explizit eingeplant werden, da anderenfalls die Gefahr besteht, dass auf unsystematische und intransparente Weise über die Priorität von Forschung entschieden wird.

Da in einer Pandemie oft zeitkritisch gearbeitet werden muss, viele Ressourcen und Personen schnell gebunden sind und es vermehrt zu Mehrfachbelastungen von Expert*innen kommt, muss formal sichergestellt werden, dass Priorisierungen durchgeführt werden können. Dazu zählen die

Finanzierung und entsprechende Qualifikation des Personals, zeitliche Kapazitäten und digitale Unterstützungstools sowie eine digitale Kollaborationsstruktur zur kontinuierlich gewährleisteten Kommunikation und Zusammenarbeit.

Bildung von stehenden Strukturen

Für eine transparente, nachvollziehbare Forschungspriorisierung sind festgelegte Strukturen für den Zeitraum der Priorisierung notwendig. Dazu zählen eine **administrative Einheit**, die einen Überblick über den gesamten Prozess behält und ein **Priorisierungs-Panel**, welches die eigentliche Priorisierung durchführt. Eine dauerhafte Etablierung dieser Strukturen ist bei fortlaufender Priorisierungsnotwendigkeit und bei der Einbettung in verstetigte Anwendungsgebiete ratsam. Vorteile davon sind klar benennbare Zuständigkeiten und Kontaktstellen für Externe, z. B. zum Einbringen von Fragestellungen.

Einbindung weiterer Perspektiven und Interessengruppen

Weitere relevante externe Interessengruppen bzw. Interessenvertretungen (in anderen Kontexten auch „Stakeholder" genannt), wie z. B. Politiker*innen, patient*innennahes Gesundheitspersonal, Public Health-Expert*innen oder Leitlinien-Gruppen, sollten in den Priorisierungsprozess eingebunden werden. Ihre Rolle im Prozess muss klar definiert werden. Eine möglichst frühzeitige Einbindung ist entscheidend, da Perspektiven und zu priorisierende Fragestellungen idealerweise aktuelle Bedarfe (auch aus der Praxis) abbilden. Eine aktive und aktuelle Tätigkeit bzw. Expertise im jeweiligen Fachbereich, aufgrund dessen die jeweiligen Personen ausgewählt wurden, ist ebenso wünschenswert. Unter hochdynamischen Pandemiebedingungen kann bzw. muss dieses Kriterium allerdings ggf. zurückgestellt werden, um eine rasche Priorisierung zu gewährleisten.

Erstellung von übergeordneten Bewertungskriterien

Die Durchführung der Priorisierung in Form eines Ratings von Fragestellungen und Themen erfordert die vorherige Definition ausgewählter Bewertungskriterien, anhand derer Forschungsfragen hinsichtlich ihrer Priorität bewertet werden. Dies stellt die Operationalisierung des Vorhabens dar und soll für ein möglichst standardisiertes und nachvollziehbares Vorgehen sorgen. Die Bewertungsdimensionen sollten sich an Gesundheits- und Versorgungszielen orientieren und auf bisheriger Forschung oder etablierten Verfahren basieren. Sie sollten für den vorherrschenden Kontext entwickelt bzw. ausgewählt werden. Eine Ergänzung um fachspezifische Bewertungsdimensionen oder die Bewertung von Fragestellungen und Themen pro Disziplin ist möglich und kann sogar dringend erforderlich sein.

4.2 Prozedurale Grundprinzipien

Demokratischer Diskurs und Ausgewogenheit

Für die Priorisierung von Fragestellungen und Themen ist ein Diskurs erforderlich, der gewährleistet, dass keine Disziplin automatisch eine dominante Rolle einnimmt oder der Priorisierungsprozess durch Zugehörigkeit der Beteiligten beeinflusst wird. Dies kann geschehen, wenn Disziplinen quantitativ

überrepräsentiert sind oder einzelne Mitglieder eine herausragende Stellung, beispielsweise durch berufsbedingte Hierarchien, innehaben. Mit einem demokratischen Diskurs ist demnach die gleichrangige Stellung aller Mitglieder gemeint. Gegebenenfalls müssen auch Priorisierungsrunden innerhalb verschiedener Disziplinen initiiert werden, wenn eine interdisziplinäre Priorisierung nicht zielführend erscheint. Beispiele für Disziplinen sind Intensivmedizin, Infektionsmedizin, Hygiene, Public Health.

Transparenz

Das Kriterium der Transparenz gilt für alle Schritte eines strukturierten Priorisierungsprozesses, sodass Entscheidungen über als prioritär bewertete Fragestellungen im Anschluss nachvollziehbar und begründbar sind. Dieses Grundprinzip beinhaltet auch die transparente Offenlegung potenzieller Interessenkonflikte aller am Priorisierungsprozess Beteiligten und ein entsprechendes Interessenkonfliktmanagement.

Kontinuierlicher Priorisierungsprozess

Während einer Pandemie ist es wahrscheinlich, dass die Priorisierung von Forschungsfragen und -themen sowohl zu Beginn als auch in regelmäßigen Abständen erneut erfolgen muss. Pandemien stellen eine akute Herausforderung für die klinische Versorgung und öffentliche Gesundheit dar, sind aber auch durch eine sich schnell wandelnde Evidenzlage, durch rasche Veränderungen im Infektionsgeschehen sowie eine Vielzahl neu auftretender gesundheitlicher und gesellschaftlicher Herausforderungen gekennzeichnet. So können neu aufkommende Fragestellungen ad hoc aufgenommen und die Priorisierung bisheriger Fragestellungen aufgrund von Änderungen in ihrer Dringlichkeit angepasst werden. Externe Interessengruppen bzw. Interessenvertretungen sollten ebenso kontinuierlich in die Priorisierung einbezogen werden.

Adaptierbarkeit des Priorisierungsprozesses

Alle in diesem Konzept beschriebenen Prinzipien und Prozesse der Priorisierung sind in hohem Maße kontextspezifisch, weswegen sie als allgemeiner Rahmen und Richtlinien verstanden werden sollten, die bei Bedarf angepasst werden können. Zu beachten ist, dass mögliche Adaptionen nicht zu Lasten der anderen Grundprinzipien umgesetzt werden und nicht die Qualität der Priorisierung beeinträchtigen sollten. Notwendige Adaptionen können sich vor allem aufgrund von zeitlicher Dringlichkeit oder organisatorischen Schwierigkeiten ergeben. Bei einer formalen Forschungspriorisierung sollten solche Abweichungen des Prozesses immer dokumentiert und begründet sein. Zu Pandemiebeginn oder in Phasen, in denen eine hohe Pandemiedynamik gegeben ist, können z. B. häufigere Priorisierungsrunden als geplant erforderlich sein, um Fragestellungen zu bewerten. In solchen hochdynamischen Phasen ist unter Umständen auch eine Vereinfachung des unter 5. beschriebenen Priorisierungsprozesses erforderlich.

5. Schritte der Priorisierung von Forschungsfragen und -themen im Kontext einer Pandemie

In diesem Kapitel wird ein 7-schrittiger Prozess zur Priorisierung von Forschungsfragen und -themen im Kontext einer Pandemie vorgestellt, bei dem die oben beschriebenen Grundprinzipien einbezogen wurden. Abbildung 1 veranschaulicht diesen schrittweisen Prozess und verdeutlicht, dass für die Priorisierung mehrere vorbereitende sowie Schritte in Rückkopplungsschleifen notwendig sind. Im Folgenden wird jeder Schritt mittels Übersichtstabelle und dazugehöriger Beschreibung dargestellt.

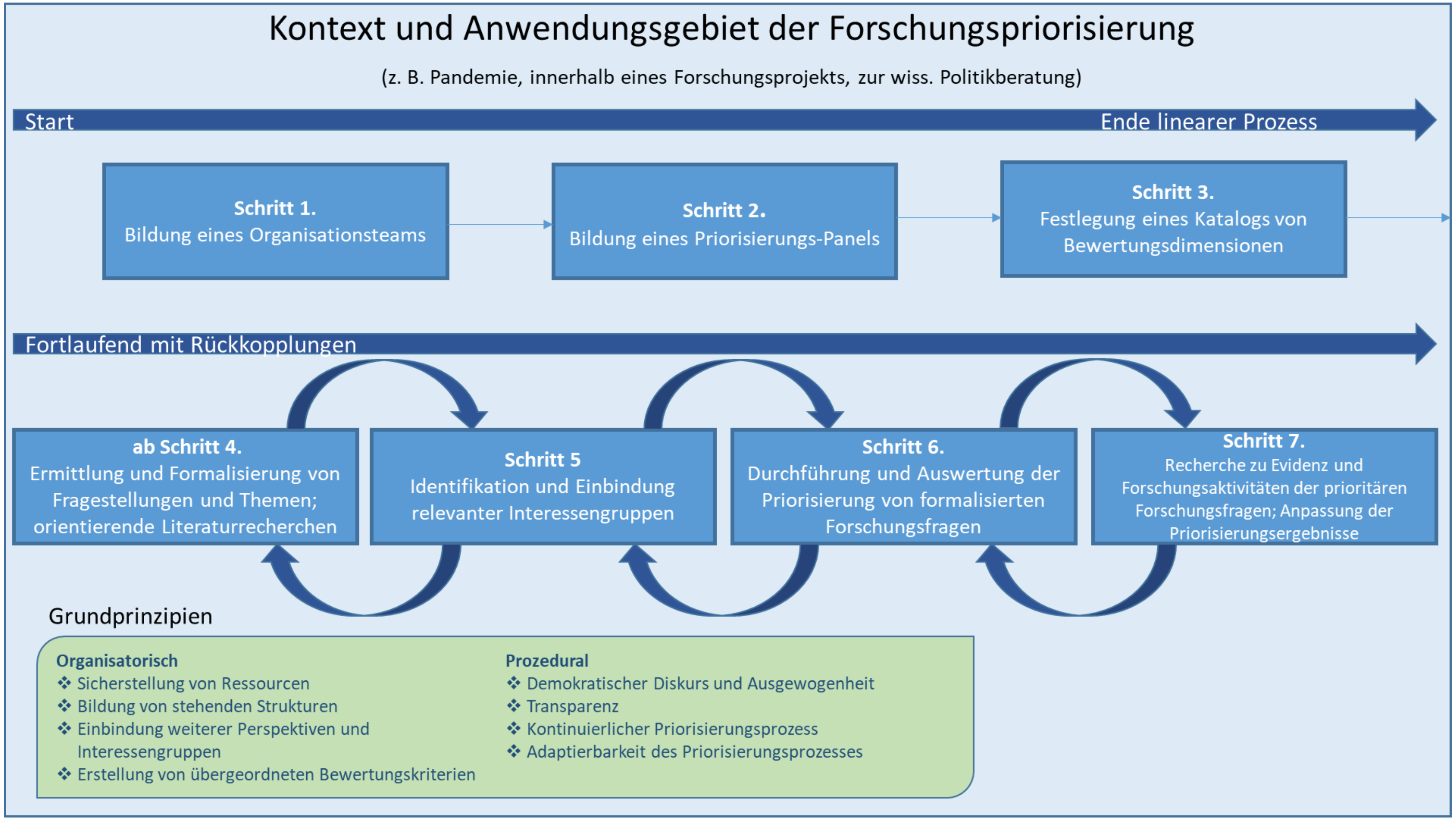

Abbildung 1. Prozess der Forschungspriorisierung

Schritt 1. Bildung eines Organisationsteams

**Ziel**	Bildung eines Organisationsteams zur fortlaufenden Koordination und zum Monitoring des Priorisierungsprozesses
**Verantwortliche**	Projektkoordinator*innen, Expert*innengremium oder sonstige befugte Personen
**Aktivitäten und weitere relevante Elemente**	• Bildung des Organisationsteams<br>• Anbindung des Organisationsteams an Strukturen des Anwendungskontextes<br>• Möglichst an einem Standort tätige Mitglieder (enge Kooperation und gemeinsame IT-Zugänge erforderlich) und schlanker Umfang<br>• Mitglieder mit Kenntnissen in Projektkoordination und gängiger Projektmanagement-Software<br>• Sehr enge Vernetzung mit Priorisierungs-Panel (siehe Schritt 2)
**Ergebnis**	Organisationsteams zur Forschungspriorisierung ist gebildet und vernetzt und die Aufgaben sind definiert

Erläuterungen zu Schritt 1

Während der COVID-19-Pandemie wurde deutlich, dass es gerade in einer so umfassenden und akuten Krise wie einer Pandemie häufig zu konkurrierenden Verpflichtungen innerhalb von Politik und Forschung kommt, sodass für den administrativen Aufwand explizit Ressourcen vorgehalten werden sollten.

Schritt 1 sieht daher die Bildung eines administrativen Teams vor, das hier als Organisationsteam betitelt wird. Ziel dieser Struktur ist es, einen Überblick über die Abläufe und Inhalte der Forschungspriorisierung zu behalten, diese zu organisieren und neutral zu begleiten. Das Organisationsteam ist *nicht* für die inhaltliche Priorisierung verantwortlich (dies macht das in Schritt 2 erläuterte Priorisierungs-Panel), sondern behält Abläufe im Blick (Monitoring) und leitet notwendige Schritte ein. Das Organisationsteam sollte gut in den Kontext der Priorisierung eingebunden sein und als Erstes oder parallel zu Schritt 2 (Bildung des Priorisierungs-Panels) etabliert werden.

Trotz des administrativen Charakters muss das Organisationsteam auch inhaltliche Kenntnisse über den Priorisierungsbereich haben, da es diejenigen Fachdisziplinen festlegt, die im Priorisierungs-Panel repräsentiert sein sollen. Dazu kann und sollte es sich auch mit weiteren Verantwortlichen wie Förderern, Wissenschaflter*innen oder Inhaltsexpert*innen austauschen und ggf. benötigte Informationen und Einschätzungen einholen. Weitere Aufgaben sind die Terminierung der Sitzungen des Priorisierungs-Panels, das Protokollieren von Meetings, die Dokumentation von Arbeitsständen sowie die Festlegung und Kontrolle von Fristen und des gesamten Prozesses. Das Team sollte einen gemeinsamen Zugriff auf die Dokumente der Priorisierung haben und diese gesammelt verwalten. Dazu gehören zu priorisierende Fragestellungen und Themen, Ergebnisse von Priorisierungsrunden oder Erklärungen von Interessenkonflikten der Mitglieder des Panels.

Beispiel NUM: Auf übergeordneter Ebene im NUM könnte das Organisationsteam in Absprache mit der NUM-Koordinierungsstelle als feste Einheit benannt werden, die dauerhaft besteht, sodass Forschungspriorisierung projekt- und infrastrukturübergreifend durchgeführt wird. Spezifischer ist geplant, dass sich eine solche Organisationseinheit im Rahmen von PREPARED in den Kernfunktionalitäten Evidenzsynthesen für vertrauenswürdige Evidenz (ESVE) und/oder an das koordinierente und bündelnde Rapid Reaction and Response- Cockpit (R3-Cockpit) wiederfindet. Ein möglicher personeller Umfang könnte bei 1-4 Personen (z. B. 2 Personen in Vollzeit mit Stellvertretung für potenzielle Ausfälle). Das Organisationsteam sollte damit ein dauerhafter Bestandteil der PREPARED-Infrastruktur sein. Da das NUM über viele beteiligte Universitätskliniken und weitere Kooperationspartner verfügt, erscheint es realistisch, dass Personen eingebunden werden können, die bereits über mehrjährige Erfahrung im Projektmanagement, in der Verwaltung oder der Projektkoordination verfügen.

Schritt 2. Bildung eines Priorisierungs-Panels

**Ziel**	Bildung eines Priorisierungs-Panels zur Durchführung der Forschungspriorisierung
**Verantwortliche**	• Sofern möglich: Organisationsteam organisiert die Panel-Bildung unter Einbezug weiterer Expert*innen<br>• Priorisierungs-Panel kann auch durch andere verantwortliche Personen innerhalb des Priorisierungskontextes gebildet werden (z. B. von Mitgliedern eines Forschungsprojekts)
**Aktivitäten und weitere relevante Elemente**	• Bildung / Benennung des Priorisierungs-Panels durch Verantwortliche<br>• Anbindung an Struktur / Netzwerk / Projekt oder sonstiges Anwendungsgebiet, jedoch über Standorte verteilt (Risikostreuung bei kurzfristigem Ausfall)<br>• Festlegung einer Panel-Leitung (z. B. drei Personen) mit festgelegter Rangfolge bei Ausfall<br>• Dokumentation einer Risikoanalyse zu konkurrierenden Verpflichtungen der Panel-Mitglieder<br>• Sicherstellung einer Doppelbesetzung für jede Fachdisziplin und für Mitglieder mit erhöhtem Risiko für konkurrierende Verpflichtungen (durch vorherige Risikoanalyse)<br>• Ausfüllen eines Letter of Intent für Panel-Mitglieder<br>• Ausfüllen eines Conflict of Interest-Formulars der Panel-Mitglieder
**Ergebnis**	Mitglieder des Priorisierungs-Panels und deren Vertretungen wurden unter Berücksichtigung von abzubildenden Fachdisziplinen festgelegt

Erläuterungen zu Schritt 2

Dieser Schritt des Priorisierungsprozesses umfasst die Bildung eines fachdisziplinübergreifenden Priorisierungs-Panels mit Vertreter*innen aus denjenigen Fachbereichen, welche angesichts einer Pandemie und unter den jeweiligen Rahmenbedingungen der Forschungspriorisierung, für die klinische Versorgung und öffentliche Gesundheit von besonderer Bedeutung sind. Zum Rahmen der

Forschungspriorisierung gehören Fragen wie: Zu welchem Zweck werden Forschungsfragen priorisiert? Beschränkt sich die Priorisierung auf einen bestimmten Inhalt oder methodische Forschungsschwerpunkte (z. B. nur Clinical Trials) oder handelt es sich um eine weiter gefasste Priorisierung (inhalts- oder methodenoffen)? Der Rahmen sollte die Auswahl an geeigneten Panel-Mitgliedern bzw. den einzubindenden Disziplinen informieren. Die Größe des Panels ergibt sich aus der Anzahl der zu vertretenden Disziplinen und Fachbereiche, um Handlungs- und Entscheidungsfähigkeit zu ermöglichen. Anpassungen können jedoch im Verlauf erforderlich sein.

Eine dauerhaft gewährleistete Besetzung des Panels stellt sicher, dass Priorisierungen auch dann vorgenommen werden können, wenn andere Anforderungen an den Priorisierungsprozess aufgrund einer akuten Dringlichkeit der Priorisierung zeitweise nicht oder vermindert erfüllt werden können. Dazu sollte jedes Panel-Mitglied eine oder mehrere Vertretungen benennen. Im Vorfeld der Panel-Bildung erscheint hierfür eine Risikoanalyse konkurrierender Verpflichtungen potenzieller Mitglieder sinnvoll, um die Arbeitsfähigkeit dieses Expert*innen-Gremiums auch in akuten Pandemiephasen zu gewährleisten.

Es ist von großer Bedeutung für den Prozess, dass ein Panel-Mitglied regelmäßig und wenn notwendig für Priorisierungen zur Verfügung stehen, weshalb eine Absichtserklärung in Form eines Letter of Intent ausgefüllt werden sollte. Auch Interessenkonflikte sollten schriftlich erfragt werden, um möglichst auszuschließen, dass bestimmte Themen aus Eigeninteresse bevorzugt behandelt werden.

Für eine allumfassende Aufnahme wichtiger Perspektiven und für die Einschätzung zur Relevanz von eingegangenen Fragestellungen und Themen ist außerdem von Vorteil, wenn Panel-Mitglieder gut in der Fachwelt vernetzt sind, z. B. durch eigene Mitwirkung in internationalen Methodennetzwerken oder bundesweit agierenden Gremien oder Netzwerken. Aus diesem Grund sollten die Mitglieder im pandemischen Kontext auch aktuell und aktiv in ihrem jeweiligen Fachgebiet tätig sein.

Beispiel NUM: Das Panel kann in Pandemien analog zu Schritt 1 im Rahmen von PREPARED als weiteres Modul im R3-Cockpit etabliert werden. Auch die Etablierung eines solchen Panels auf NUM-Ebene oder in weiteren Teilprojekten ist möglich. Die Organisation erfolgt idealerweise jeweils durch das in Schritt 1 beschriebene Organisationsteam und ggf. unter Einbezug weiterer Expert*innen. Im Sinne einer langfristigen „Pandemic Preparedness“ sollte erwogen werden, Priorisierungs-Panels als langfristige und verstetigte Strukturen zu etablieren, die auch in inter-pandemischen Phasen aufrechterhalten werden und angesichts zukünftig auftretender Pandemien oder weiterer Krisensituationen schnell reagieren und eine Priorisierung von Forschungsfragen und -themen zügig vornehmen können.

Potenzielle Panel-Mitglieder können innerhalb des NUM, aus weiteren existierenden wissenschaftlichen Netzwerken (z. B. Deutsches Netzwerk Evidenzbasierte Medizin oder anderer Bereiche), med. Fachgesellschaften oder existierenden Leitlinien-Gruppen sowie über Netzwerke oder Einrichtungen aus dem ÖGD (z. B. Netzwerk Gesunde Städte der Weltgesundheitsorganisation [WHO], Netzwerk Gesundheitsämter) rekrutiert werden.

Für die COVID-19-Pandemie wurden u. a. methodisch-inhaltliche Kompetenzen aus Public Health, evidenzbasierter Medizin (EbM), Modellierung und klinischer sowie pflegewissenschaftlicher

Primärforschung benötigt. Klinisch sind in einer Pandemie voraussichtlich die Fachbereiche Infektiologie, Intensivmedizin, Hygiene und Infektionsprävention, Allgemeinmedizin sowie Psychiatrie und Pflegewissenschaften relevant. Der öffentliche Gesundheitsdienst sollte durch Vertreter*innen der Bundes-, Landes- und der kommunalen Ebene abgebildet werden. Bei der Bildung des Panels ist zu beachten, dass eine Person auch mehrere Aspekte abbilden kann (z. B. klinisch und wissenschaftlich Tätige). Dies sind die Disziplinen, die für ein auf übergeordneter Ebene stehendes Priorisierungs-Panel im NUM vorgeschlagen werden. Auch für Priorisierungen innerhalb von PREPARED kann durch eine weit verzweigte Vernetzung von Kooperationspartnern ein solches Panel aufgestellt werden. Für eine Priorisierung in einzelnen Forschungsprojekten, z könnte voraussichtlich ein Panel mit weniger Fachdisziplinen ausreichen.

Schritt 3. Festlegung eines Katalogs von Bewertungskriterien

**Ziel**	Erstellung oder Festlegung von Bewertungskriterien zur Durchführung der Priorisierung
**Verantwortliche**	• Organisationsteam<br>und<br>• Priorisierungs-Panel<br>• Ggf. weitere einzubindende Mitarbeitende, z. B. zu Recherchezwecken
**Aktivitäten und weitere relevante Elemente**	• Recherche nach passenden Kriterien für die Priorisierung<br>• Überprüfung der Kriterien auf notwendige Anpassungen an den Kontext und das Anwendungsgebiet<br>• Ggf. Hinzunahme oder Aussparung von Kriterien<br>• Gemeinsame Entscheidung über den Eingang von Kriterien zur Priorisierung<br>• Pilotierung der Kriterien in einer beispielhaften Priorisierung<br>• Erstellung von Erläuterungen und Fallbeispielen zu den einzelnen Kriterien<br>• Ggf. Einteilung in einzelne Bewertungskriterien und übergeordnete Bewertungsdimensionen<br>• Ggf. Anpassungen pro Disziplin oder Fachbereich
**Ergebnis**	Anwendungsfreundlicher Kriterienkatalog zur Durchführung der Forschungspriorisierung inklusive Bewertungsmanual mit detaillierten Erklärungen für verschiedene Bewertungskriterien und Fallbeispielen wurde erstellt

Erläuterungen zu Schritt 3

Möglichst zügig nach der Bildung des Organisationsteams und des Priorisierungs-Panels sind Bewertungskriterien festzulegen, anhand derer später eine Einschätzung für jede einzelne Fragestellung vorgenommen werden kann. Dieser Schritt ist recht aufwändig, da neben der Recherche und Auswahl geeigneter Kriterien auch die Erstellung eines Manuals und / oder ein Testlauf für die Anwendung vorgesehen ist. So soll die Verständlichkeit und Anwendbarkeit der Materialien sichergestellt werden.

Je nach Pandemiephase oder sonstiger Krisensituation kann ein Vorgehen nach der Delphi-Methode innerhalb des Priorisierungs-Panels sinnvoll sein, um einen Konsens zur Auswahl zu schaffen. Alternativ und ausgehend von Vorerfahrungen aus der COVID-19-Pandemie (vgl. Projekt CEOsys) kann die Festlegung basierend auf der bisherigen Forschung (z. B. El-Harakeh et al., 2019) oder etablierten Ansätzen bzw. einer Kombination daraus ebenfalls zielführend sein. Ein Beispiel für ein solches Verfahren stellt das UpPriority-Tool zur Standardisierung des Priorisierungsprozesses bei der Aktualisierung klinischer Leitlinien dar (Sanabria et al., 2020). Darüber hinaus existieren weitere Methoden, auf die aufgrund ihrer Komplexität und Fülle hier nicht weiter eingegangen wird. Wann immer möglich, sollten jedoch praxisbewährte oder validierte Bewertungskriterien herangezogen werden, soweit sich diese in der internationalen Literatur für den jeweiligen Anwendungsbereich der Priorisierung identifizieren lassen. Diese können auch adaptiert werden, um abweichende Gegebenheiten zu adressieren (z. B. andere Art des Gesundheitssystems). Insbesondere bei Auftreten eines unbekannten Erregers und mangelnder Forschungslage müssen Bewertungskriterien anhand von Expert*inneneinschätzungen festgelegt werden. Die Erstellung von Templates für solche Kriterien sollte forciert werden, um im Akutfall handlungsfähig zu sein. Die Templates sollten regelmäßig überprüft und angepasst werden. Darüber hinaus sollten sie so generisch wie möglich sein, ohne Besonderheiten der einzelnen Fachbereiche zu vernachlässigen.

Für die Auswertung kann es zur Orientierung sinnvoll sein, den Kriterien übergeordnete Bewertungsdimensionen zuzuordnen. So können Kriterien sinnvoll zusammengefasst werden. Orientiert am UpPriority-Tool (Sanabria et al., 2020) kann es sich bei übergeordneten Dimensionen beispielsweise um Aspekte handeln wie die Verfügbarkeit von (neuen) Primärstudien mit relevanten Erkenntnissen, die methodische Anwendbarkeit der Fragestellung oder das Interesse der Nutzer*innen (z. B. Wissenschaftler*innen, Entscheidungsträger*innen, Patient*innen, Allgemeinbevölkerung). Auch das AWMF-Regelwerk (AWMF, 2020) nennt mehrere Kriterien, wie die Häufigkeit des Versorgungsaspekts (z. B. Prävalenz, Inzidenz), das Optimierungs- und / oder Verbesserungspotenzial der Versorgungsqualität oder die Krankheitslast (individuell, bevölkerungsbezogen).

Beispiel NUM: Auf Projektebene wurde in CEOsys ein Bewertungskatalog für die Priorisierung von Forschungsfragen für Evidenzsynthesen erstellt. Die Methodik für die anschließende Bearbeitung der Fragen war demnach schon festgelegt und die Priorisierung hatte den Zweck, zu entscheiden, welche Fragen in welcher Reihenfolge bearbeitet werden sollten. Das dazugehörige Template mit einer Übersicht der in CEOsys verwendeten Bewertungskriterien befindet sich im Anhang.

Bei der Etablierung verstetigter Priorisierungsstrukturen wäre auch die zentrale Ablage eines erstellten Kriterienkatalogs in der dafür vorgesehenen NUM-Cloud als Teil des NUM Hubs sinnvoll. Sowohl ein übergeordnetes Priorisierungs-Panel als auch zusätzliche Panels auf Forschungsebene könnten hierdurch sowohl auf bestehende Materialien zugreifen als auch selbst Ordnerstrukturen anlegen und Dokumente erstellen, hochladen und bearbeiten.

Schritt 4. Ermittlung von Fragestellungen und Themen zur Priorisierung und Formalisierung von Forschungsfragen

**Ziel**	Sammlung von Fragestellungen und Themen zur Priorisierung und Formalisierung von Forschungsfragen
**Verantwortliche**	• Einreichende Personen oder Institutionen<br>• Organisationsteam<br>• Priorisierungs-Panel<br>• Ggf. Einbezug weiterer Mitarbeiter*innen
**Aktivitäten und weitere relevante Elemente**	• Festlegung von Wegen und Zuständigkeiten für u.g. Aktivitäten<br>• Erstellung und Nutzung von Templates zur Einreichung von Fragestellungen und Themen<br>• Organisationsteam verwaltet alle eingehenden Fragestellungen und Themen und fasst Dopplungen zusammen<br>• Operationalisierung der Fragen: Berücksichtigung von Prinzipien der Erstellung von Forschungsfragen, z. B. Nutzung von Frameworks für Forschungsfragen (z. B. PICO: Population, Intervention, Comparator, Outcomes)<br>• Ggf. schnelle, orientierende Literaturrecherchen zu den nun operationalisierten Forschungsfragen<br>• Fragestellungen und Themen erreichen das Panel und werden nur unter bestimmten Bedingungen vom Panel selbst eingebracht
**Ergebnis**	Wege, Zuständigkeiten und Legitimierung über den Eingang von Fragen und Themen, die Vorselektion und Formalisierung von Forschungsfragen für die Priorisierung wurden festgelegt; Pool an zu priorisierenden Fragestellungen ist entstanden und wird fortlaufend aktualisiert

Erläuterungen zu Schritt 4

Ab diesem Schritt ist der Priorisierungsprozess nicht mehr linear, sondern inklusive Rückkopplungen zu verstehen, nachdem die dauerhaft bestehenden Strukturen aus Schritt 1-3 festgelegt wurden. Das Auftreten von Forschungsfragen findet in einer Pandemie nicht einmalig statt, sondern kontinuierlich und aufgrund akuter, sich schnell ändernder Kontexte wahrscheinlich mit einer höheren Frequenz.

Fragestellungen und Themen erreichen das Panel vorrangig von extern, d. h., das Panel selbst bringt primär keine Fragestellungen und Themen ein. Sollten während der Operationalisierung oder an anderen Punkten im Prozess dennoch Fragen innerhalb des Panels identifiziert werden, sollte ein explizites Interessenskonfliktmanagement hierfür angewandt werden. Fragen und Themen können z. B. aus der Wissenschaft, der Politik und der Gesundheitsversorgung (innerhalb der NUM-Community) und bei der Etablierung entsprechender Strukturen auch aus der Bevölkerung kommen. Nur nachgeordnet sollte das Panel selbst Fragen einfließen lassen dürfen, um die Objektivität in der Priorisierung sicherzustellen. Für die Einreichung sollten mögliche Wege und Berechtigungen festgelegt werden. Die Etablierung von Kontaktformularen und Ansprechpersonen wird hierfür empfohlen.

Einreichungen werden dann im Sinne eines induktiv-deduktiven Ansatzes an das Priorisierungs-Panel herangetragen. Induktiv-deduktiv meint hier, dass Fragestellungen sowohl induktiv aufgrund von akut auftretenden Ereignissen und Beobachtungen im pandemischen Kontext aufkommen können (theoriebildend) als auch durch bereits bestehende Annahmen, Zusammenhänge und Theorien (theoriegeleitet). Beispiele für einreichende Personen / Institutionen sind im ambulanten oder stationären Setting tätige Kliniker*innen, Forschungsgruppen mit thematischer Ausrichtung im Bereich der interessierenden Fragestellung, med. Fachgesellschaften, bestehende Leitlinien-Gruppen, Fördereinrichtungen oder Politiker*innen. Die Öffnung hin zur Einreichung von Fragen durch die Bevölkerung sollte ebenfalls diskutiert werden, jedoch von der jeweiligen Pandemiephase abhängig gemacht und in Akutphasen zum Zweck einer zügigen Priorisierung eher zurückgestellt werden.

Anschließend müssen Fragestellungen und Themen in das Format einer Forschungsfragestellung gebracht werden, bevor eine Priorisierung möglich ist. Forschungsprojekte in der COVID-19-Pandemie haben gezeigt, dass dieser Schritt oft notwendig ist und Zeit beansprucht. Die präzise Formulierung einer Fragestellung ist allerdings unerlässlich für eine Beurteilung der Forschungspriorität, da die nachfolgende Forschung auf diesen Fragestellungen aufbaut. Die Zuständigkeit für diese Aufgabe muss ebenfalls zu Beginn festgelegt werden und sollte von wissenschaftlichem Personal durchgeführt werden.

Folgende Frameworks zur Generierung von Forschungsfragen sind beispielhaft hierfür:

- PICO: Population, Intervention, Comparator, Outcomes
- PECO: Population, Exposure, Comparator, Outcomes
- PIRT: Population, Index test, Reference test, Target condition
- PICOTS: Population, Index prognostic factor or model, Comparator factor or model, Outcomes, Timing of prognostication, Setting of prognostication

Das Organisationsteam behält den Überblick über identifizierte Fragestellungen. Dafür muss eine entsprechende digitale Infrastruktur bereitstehen, in der auch der Bearbeitungsstatus einsehbar ist. Bei der Erfassung von Fragestellungen ist es ideal, wenn anstelle allgemeiner Themenbereiche (z. B. Tragen von medizinischem Mund-Nasen-Schutz, Impfung etc.) schon möglichst konkret formulierte Fragen eingereicht werden, um den späteren Aufwand zu verringern. Hierfür kann ein entsprechendes Template unterstützen, welches alle erforderlichen Informationen zur Formulierung einer klaren Forschungsfrage abfragt. Je nach Anzahl der eingehenden Fragen und Themen ist es möglicherweise notwendig, eine Vorselektion zu treffen, da eine formale Operationalisierung und anschließende Priorisierung einer großen Menge an Fragen zeitliche Ressourcen übersteigen könnte.

Weiterhin kann eine schnelle, orientierende Literaturrecherche zu operationalisierten Fragen notwendig sein, um eine Einschätzung der Relevanz und Dringlichkeit treffen zu können. Auch hierfür sollte Expertise vorherrschen und die Zuständigkeit über die Durchführung dieser Recherchen geklärt werden.

Beispiel NUM: Auch bei diesem Schritt stellt die NUM Cloud eine gute Möglichkeit dar, identifizierte Fragestelllungen digital zu bündeln und zu verwalten. Das Organisationsteam könnte über ein Kontaktformular auf der NUM Webseite oder auf der jeweiligen Projektwebseite erreichbar sein,

welches schon entsprechend der festgelegten Berechtigungen von Personengruppen gestaltet sein sollte. Vorstellbar ist auch die Öffnung dieser Möglichkeit nur für den internen Bereich des NUM. Die Weiterleitung an das Priorisierungs-Panel geschieht anschließend, z. B. über die NUM-Cloud oder einen E-Mailverteiler. In regelmäßigen Treffen geschieht die ggf. notwendige Vorselektion und Formalisierung von Forschungsfragen. Infrastrukturen wie PREPARED können durch ihre Nähe zur Politik, Selbstverwaltung und weiteren Akteuren sicherstellen, dass Priorisierungsvorhaben bekannt und Wege zur Einreichung von Fragestellungen etabliert sind.

Schritt 5. Frühzeitige Identifikation und Einbindung relevanter Perspektiven und Interessengruppen

**Ziel**	Einbindung externer, möglichst repräsentativer Interessengruppen zur Berücksichtigung relevanter Perspektiven
**Verantwortliche**	• Priorisierungs-Panel<br>• Unterstützung durch Organisationsteam<br>• Vertreter*innen ausgewählter Interessengruppen
**Aktivitäten und weitere relevante Elemente**	• Identifikation von weiteren benötigten Perspektiven<br>• Ableitung einzubindender Interessengruppen und Versendung von Anfragen an Institutionen, Netzwerke oder Einzelpersonen<br>• Bestimmung der Rolle hinzugezogener Interessengruppen<br>• Ggf. Ergänzung weiterer relevanter Fragestellungen<br>• Letter of Intent für externe Beteiligte<br>• Conflict of Interest-Formular für externe Beteiligte<br>• Nutzung von Templates zur Einreichung von Fragen und Themen, zur Vorselektion und zur Formalisierung von Forschungsfragen ratsam
**Ergebnis**	Übersicht über relevante Perspektiven außerhalb des Priorisierungs-Panels, erfolgreiche Rekrutierung daraus abgeleiteter Vertreter*innen von Interessengruppen mit definierter Rolle im Prozess und ggf. Einbezug weiterer Forschungsfragen in den Pool von zu priorisierenden Forschungsfragen

Erläuterungen zu Schritt 5

Anhand des Anwendungsgebiets und der eingereichten und formalisierten Fragestellungen sowie durch Diskussionen entscheidet das Priorisierungs-Panel in Schritt 5, welche weiteren Interessengruppen zusätzlich für die Berücksichtigung wichtiger Perspektiven, das Einbringen weiterer Fragen und Themen oder sogar für die Priorisierung selbst relevant sind und frühzeitig eingebunden werden sollten. Im Anschluss identifiziert das Priorisierungs-Panel diese Interessengruppen und entsprechende Vertreter*innen, beispielsweise vermittelt über die AWMF oder existierende Interessengruppen wie Betroffenenverbände. Das Organisationsteam unterstützt bei organisatorischen Aufgaben, führt ggf. Recherchen zu potenziellen Vertreter*innen durch und versendet Anfragen zur Beteiligung.

Orientiert an der Zusammensetzung einer Leitlinien-Gruppe nach dem AWMF-Regelwerk (AWMF, 2020) können externe Interessengruppen beispielsweise folgende Kriterien erfüllen:

- Erfahrene Anwendende aus der klinischen Praxis (z. B. Ärzt*innen, Pflegekräfte, Physiotherapeut*innen, Psycholog*innen, Angehörige weiterer Berufsgruppen) und Patient*innen / Betroffene bzw. Bürger*innen
- Repräsentanz von professioneller und wissenschaftlicher Expertise sowie der Perspektive / Präferenzen von Betroffenen in den identifizierten Themenbereichen

Allgemein sollten einbezogene Interessengruppen als solche gelten, wenn sie entweder Verantwortungsträger*innen, an Prozessen oder Entscheidungen beteiligt oder von diesen betroffen sind. In einer Pandemie kann dies dazu führen, dass sehr viele Personen, Gruppen oder Netzwerke als potenzielle Interessengruppen legitimiert werden können. Hier gilt es, den Einbezug zu begründen.

Die Bereitschaft und zeitliche Kapazitäten sollten vorab geklärt sowie Rollen und Aufgaben klar definiert werden (z. B. Teilnahme an Meetings). Zudem sollte auch festgelegt werden, ob lediglich Stellungnahmen oder auch konkrete Fragestellungen eingebracht werden dürfen, die eine formale Priorisierung durchlaufen oder ob die Vertreter*innen sogar selbst bei der formalen Priorisierung beteiligt sind. Das Organisationsteam bereitet im Anschluss gemeinsame Sitzungen zwischen dem Panel und relevanten Interessengruppen vor bzw. lädt einzelne Vertreter*innen zu Sitzungen ein, um weitere relevante Fragestellungen zu identifizieren. Diese zusätzlich identifizierten Fragestellungen werden ebenfalls durch das Priorisierungs-Panel oder gemeinsam formalisiert.

Zusätzlich zu einer möglichst frühen Einbindung relevanter Interessengruppen sollten diese zusätzlich im weiteren Verlauf eingebunden werden, da durch neue Themen und Fragen auch neue Bedarfe entstehen können.

Beispiel NUM: Im Kontext des NUM und einer Pandemic Preparedness ist durch die große Bandbreite aufkommender Themen und beteiligter Netzwerkpartner auch die Anzahl potenzieller Interessengruppen hoch. Vermutlich sind viele davon bereits selbst in das NUM eingebunden und können über bestehende Kommunikationskanäle gut erreicht werden. Die geplante agile PREPARED-Governance kann hier ebenfalls eine Schlüsselrolle einnehmen. Durch die Zugehörigkeit zum NUM ist von einer regen Beteiligung der Interessengruppen auszugehen. Eine Auflistung beteiligter Standorte befindet sich auf der NUM-Webseite (netzwerk-universitaetsmedizin.de).

Für die Forschungspriorisierung innerhalb des NUM und insbesondere von PREPARED nehmen politische Interessenvertretungen voraussichtlich eine besonders relevante Rolle ein, da u. a. die wissenschaftliche Politikberatung im Fokus steht. Von einer Beteiligung an der Durchführung der Priorisierung von formalisierten Forschungsfragen (Schritt 6) würde aufgrund von möglichen Interessenskonflikten allerdings abgeraten werden. Die Einbindung sollte hier eher in der Einreichung von Fragen, Etablierung von Prozessen und einbringen wichtiger, sonstiger Aspekte liegen.

Schritt 6. Durchführung und Auswertung der Priorisierung von formalisierten Forschungsfragen

**Ziel**	Durchführung eines Ratings zur Priorität der formalisierten Forschungsfragen anhand festgelegter Bewertungskriterien und daraus resultierende Bildung von Rangfolgen
**Verantwortliche**	• Organisationsteam (formelle Organisation)<br>• Priorisierungs-Panel<br>• Ggf. weitere extern eingebundene Interessengruppen
**Aktivitäten und weitere relevante Elemente**	• Einschätzung der Priorität für jede Forschungsfrage basierend auf den festgelegten Bewertungskriterien bzw. -dimensionen<br>• Erstellung einer oder mehrerer Ranglisten von Forschungsfragen basierend auf diesen Bewertungen<br>• Offene Felder für Begründungen und Ausführungen abseits der festgelegten Kriterien<br><br>Einige, mögliche Einteilungen:<br>• Eintrag der Fragestellungen in eine Priorisierungsmatrix<br>• Getrennte Ranglisten für verschiedene Themenbereiche oder Fachdisziplinen<br>• Gesamtrangliste mit verschiedenen Disziplinen<br>• Getrennte Ranglisten nach anzuwendender Forschungsmethodik, sofern diese schon bestimmt wird (Modellierungen, Evidenzsynthesen, klinische Studie etc.)<br>• Template (z. B. Dokument, Survey), das Forschungsfragen und Bewertungskriterien beinhaltet<br>• Template zur Dokumentation des Ratings pro Forschungsfrage
**Ergebnis**	Nachvollziehbare Priorisierung von Forschungsfragen, die mittels Rangliste(n) dargestellt wird

Erläuterungen zu Schritt 6

Unter Schritt 6 erfolgt das Rating der formalisierten Forschungsfragen in Form von Einzelbewertungen der Panel-Mitglieder anhand der Bewertungskriterien bzw. -dimensionen und in gemeinsamen Sitzungen zur weiteren Diskussion. Hierzu sollte der Bewertungskatalog mit Kriterien und Erläuterungen neben dem Panel auch den externen Interessengruppen, falls diese einbezogen werden, im Vorfeld zur Verfügung gestellt werden. Auch eine Einweisung (z. B. wichtige Hinweise zum Antwortformat und Fallbeispiele) erscheint sinnvoll. Gegebenenfalls kann das Rating auch nur auf diejenigen Panel-Mitglieder begrenzt werden, die z. B. basierend auf einer vorausgehenden Selbsteinschätzung, besondere Expertise für die spezifische Forschungsfrage aufweisen.

Die Bewertung der Forschungsfragen erfolgt zunächst in anonymisierter und verblindeter Form und wird von jeder abstimmungsberechtigten Person durchgeführt. Die Verwendung eines Antwortformats auf Intervallskalenniveau (z. B. 7-stufige Likert-Skala von 1-7; 1 = sehr niedrig bis 7 = sehr hoch) hat sich beispielsweise bei der Priorisierung von Forschungsfragen für die Aktualisierung

klinischer Leitlinien durchgesetzt (z. B. Sanabria et al., 2020). Diese Einteilung kann jedoch verändert werden.

Die Ergebnisse des Ratings werden durch das Organisationsteam zusammengetragen, gesichtet und ausgewertet. Offene Fragen und Anmerkungen können in anschließender Sitzung geklärt und Ausreißer oder Extremwerte diskutiert werden, um einen einheitlichen Bewertungsmaßstab sicherzustellen. Basierend auf den verschiedenen Bewertungskriterien resultiert pro Frage eine Gesamteinschätzung (z. B. durch Mittelwertbildung), sodass als Resultat eine Rangliste der Fragen erstellt werden kann. Hierbei kann auch die Erstellung getrennter Ranglisten für verschiedene Themenbereiche bzw. Disziplinen (z. B. klinische Versorgung und Public Health) oder für verschiedene anzuwendende Forschungsmethoden sinnvoll sein, falls eine Ressourcenallokation Gegenstand der Priorisierung ist. Auch andere Darstellungsformen für die Ergebnisse des Ratings (z. B. Priorisierungsmatrix mit Darstellung aller o. g. Dimensionen) sind denkbar.

Beispiel NUM: Ein Priorisierungs-Panel des NUM – z.B. modular an das PREPARED Cockpit angebunden, könnte wie oben beschrieben die vorgehaltenen Strukturen nutzen und darüber auch die formale Priorisierung organisieren. Der Bewertungskatalog und seine Erläuterungen könnten hinterlegt und für jedes Mitglied ein Template mit den zu bewertenden formalen Forschungsfragen zum Ausfüllen vorbereitet werden. Zunächst würde sich jedes Panel-Mitglied sein Template herunterladen und offline bis zu einem festgelegten Termin ausfüllen, um die unabhängige Bewertung zu garantieren. Anschließend könnten die ausgefüllten Dateien wieder in der NUM-Cloud zur weiteren Auswertung und Grundlage für folgende Diskussionen abgelegt werden. So entstehen priorisierte Forschungsfragen für einen festgelegten Anwendungsbereich. Auf übergeordneter Ebene könnten so beispielsweise notwendige nächste Projekte oder Anwendungsfälle für bestehende Projekte abgeleitet werden. Im Rahmen von PREPARED ist eine Expert*innengruppe geplant, die Fragestellungen aus einem Gremium bestehend aus Vertreter*innen aus Politik und Selbstverwaltung des Gesundheitswesens erhält und diese priorisiert. Hierfür könnten der hier beschriebene Prozess sowie die NUM-Strukturen genutzt werden.

Schritt 7. Recherche zu Evidenz und Forschungsaktivitäten zu den jeweiligen Fragestellungen und Anpassung der Priorisierungsergebnisse

**Ziel**	Erfassung des Forschungsstands zu priorisierten Forschungsfragen
**Verantwortliche**	• Eingebundene wissenschaftliche Mitarbeiter*innen<br>und / oder<br>• Organisationsteam<br>und / oder<br>• Priorisierungs-Panel: hier direkt vertretene bzw. mit dem Panel assoziierte und extern beauftragte Methodiker*innen<br>oder<br>• Ggf. können Expert*innen der Literaturrecherche hinzugezogen werden (Informationsspezialist*innen oder Bibliothekar*innen)
**Aktivitäten und weitere relevante Elemente**	• Vorrecherche und Sichtung der Literatur zu Forschung, weiteren aktuellen (und geplanten) Forschungsaktivitäten sowie zu politischem und gesellschaftlichem Diskurs der priorisierten Forschungsfragen<br>• Dokumentation der Rechercheergebnisse mit Angabe von genutzten Datenbanken und Quellen<br>• Ggf. Anpassung der Priorisierungsergebnisse, je nach Ergebnis der Recherche: Reduktion der Anzahl von Fragen, stärkere Spezifizierung oder Veränderung der Rangfolge von Fragen
**Ergebnis**	Dokumentierter Forschungsstand zu priorisierten Forschungsfragen und ggf. Anpassung der Priorisierung

Erläuterungen zu Schritt 7

Schritt 7 der Priorisierung beschreibt eine Vorrecherche zur existierenden Evidenz, zu geplanten oder laufenden Forschungsvorhaben und zu aktuellen gesellschaftlichen und / oder politischen Diskursen bzgl. der priorisierten Forschungsfragen. Diese Recherche sollte zu diesem Zeitpunkt keine allumfassende, zeitaufwändige Suchstrategie beinhalten, wie z. B. in systematischen Übersichtsarbeiten üblich, da durch die Vielzahl an in den Priorisierungsprozess eingebundenen Disziplinen und Interessengruppen davon auszugehen ist, dass ein gewisser Kenntnisstand vorhanden ist und in Pandemien eher Zeitnot vorherrscht. Vielmehr geht es darum, als zusätzlichen Schritt nach Abschluss der eigentlichen Priorisierung das aktuelle Geschehen zu priorisierten Forschungsfragen abzubilden, welches sich in Pandemien sehr schnell wandeln kann. Dies kann entweder zu einer erneuten Einschätzung durch das Priorisierungs-Panel (und Interessengruppen) zu den priorisierten Forschungsfragen führen oder diese bestätigen. Hierfür könnten auch Institutionen und Netzwerke, die auf schnelle Literaturrecherche spezialisiert sind, beauftragt werden.

Durch Schritt 7 kann die Anzahl und Rangfolge der priorisierten Forschungsfragen noch einmal verändert werden, um Redundanzen in der Forschungslandschaft zu vermeiden. D. h., die Ergebnisse der eher unsystematischen Vorrecherche können als ein Bewertungskriterium i.S. einer Rückkopplung (vgl. Abb. 1) auch in das Priorisierungs-Rating (siehe Schritt 6) einfließen. Schritt 7 ist insbesondere dann relevant, wenn erwartet wird, dass bereits (internationale) Forschung initiiert wurde und ggf.

sogar verwertbare Ergebnisse vorliegen oder wenn ein akuter gesellschaftlicher / politischer Bedarf absehbar ist. In besonders zeitkritischen Situationen muss auf Schritt 7 verzichtet werden.

Für die weitere Nutzung nach der Priorisierung von Forschungsfragen kann durch die Vorrecherche bereits antizipiert werden, mit welcher Methodik die Forschungsfrage bearbeitet werden kann.

NUM-Beispiel: Durch die Vernetzung innerhalb des NUM und der daraus resultierenden Expertise, auch im Bereich der systematischen Literaturrecherche, kann dieser Schritt bei rechtzeitiger Einbindung und Beauftragung rasch umgesetzt werden. Insbesondere ist innerhalb von PREPARED eine Struktur geplant, die zügige Literaturrecherchen und Evidenzsynthesen inklusive der Ableitung von Handlungsempfehlungen umsetzen soll. Diese Struktur und die darin enthaltenen Institute können Schritt 7 ausführen. Unabhängig davon kann bei jedem Priorisierungsprozess darauf geachtet werden, dass entsprechendes Personal für Schritt 7 eingeplant wurde, welches z. B. auch Teil des Organisationsteams sein kann.

Regelmäßige Wiederholung der Schritte 4-7 im dynamischen Pandemiegeschehen

Insgesamt sollte die Priorisierung möglichst keinen linearen Prozess darstellen, sondern die Schritte 4 bis 7 – in Abhängigkeit von vorhandenen Ressourcen – in einem regelmäßigen Rhythmus oder nach Bedarf wiederholt werden. Auf diese Weise lassen sich zeitnah neue Fragestellungen von hoher Aktualität und Relevanz sowie notwendige einzubeziehende Perspektiven und Interessengruppen identifizieren. Fragen sollten kontinuierlich an das Organisationsteam und das Panel herangetragen werden können und Frequenzen für Priorisierungsrunden bei Bedarf angepasst werden. Höhere Frequenzen sind z. B. bei einem hohen Aufkommen an neuen Fragen, aufgrund von unvorhergesehenen Ereignissen (z. B. Infektionsausbruch in Gesundheitseinrichtungen) oder aufgrund politischer Entscheidungsunsicherheit notwendig.

Darüber hinaus besteht die Option, dass bereits priorisierte Forschungsfragen, deren Bearbeitung jedoch noch nicht gestartet ist, im weiteren Verlauf zurückgestellt oder vorgezogen werden, sollten neue, wichtige Forschungsfragen aufkommen. Hierfür ist es erforderlich, dass das Organisationsteam den Bearbeitungsstand genau dokumentiert und das Priorisierungs-Panel darüber informiert. Diese **Rückkopplungsprozesse** erlauben eine schnelle Reaktion auf kurzfristige Änderungen in der Dringlichkeit von Fragestellungen. Zudem können im Anschluss an die Priorisierung begrenzte Ressourcen angemessen eingesetzt werden.

6. Ausblick

6.1 Einschätzung zum zeitlichen Ablauf des Priorisierungsprozesses

Der hier beschriebene Priorisierungsprozess hängt in seinem zeitlichen Ablauf davon ab, inwiefern bestehende Strukturen für die Bildung der inhärenten Gremien bestehen, welche zeitlichen Anforderungen an die Strukturen gestellt werden, welchen Stellenwert die Forschungspriorisierung bei Mitgliedern der Gremien hat, wie hoch der Grad der Standardisierung der einzelnen Schritte und Arbeitsmaterialien ist und nicht zuletzt vor allem davon, wie viele Fragestellungen und Themen an das Priorisierungs-Panel herangetragen werden.

Eine pauschale Zeitangabe über die Dauer des Prozesses kann daher nicht getroffen werden. Vielmehr sollte das weiterführende Ziel eine Pilotierung und Implementierung des Prozesses und der damit einhergehenden Strukturen sein. In weiteren Schritten sollten die o. g. Templates und digitale Kommunikationsstrukturen angelegt werden, sodass sie für weitere Priorisierungskontexte nutzbar sind und nur noch angepasst werden müssen. Sind die Schritte zur Bildung des Organisationsteams, der Bildung des Priorisierungs-Panels und die Erstellung von Bewertungskriterien absolviert und ist die Zusammenarbeit einmal erprobt, sollte der Prozess je nach Adaption einige Stunden bis Tage andauern.

Es ist zu betonen, dass der Prozess kontinuierlich fortgesetzt werden sollte, sodass neue Forschungsfragen laufend in die Rangliste(n) und / oder die Priorisierungsmatrix eingefügt werden können, und dass er außerdem adaptiv gestaltet werden sollte. Dies bedeutet, Schritte können angepasst oder sogar weggelassen werden, sollte dies erforderlich sein. Auch hier gilt, dass diese möglichen Adaptionen des Prozesses und der allgemeinen Grundprinzipien erprobt werden sollten. Voraussetzung für eine schnelle Priorisierung ist die Verfügbarkeit der Panel-Mitglieder oder ihrer benannten Vertretungen. Vor diesem Hintergrund wäre z. B. die Anpassung vorstellbar, dass nur ein gewisser Prozentsatz an Panel-Mitgliedern am Prozess beteiligt sein muss oder bei fehlender Reaktion trotzdem Priorisierungen vorgenommen werden dürfen.

6.2 Schritte nach der Priorisierung von Forschungsfragen

Im Anschluss an den Priorisierungsprozess müssen Ressourcen schließlich in ausreichendem Maße zur Verfügung stehen, um die als dringlich priorisierten Fragestellungen, die thematisch stark variieren können, auch bearbeiten zu können. Erfolgt die Priorisierung zum Zweck der Bearbeitung von Fragestellungen innerhalb eines Netzwerks wie dem NUM oder hier etablierten kleineren Teilinfrastrukturen oder -netzwerken (vgl. PREPARED), sollten die priorisierten Fragestellungen, die sich ableitende Forschungsmethodik zur Bearbeitung (z. B. Evidenzsynthese, qualitative Experteninterviews, Modellierungen) und deren Bearbeitungsstatus im Nachgang der Priorisierung dokumentiert und für alle Mitarbeitenden des Netzwerks einsehbar sein. Sind Ressourcen zur gleichzeitigen Bearbeitung mehrerer dringlicher Fragestellungen innerhalb dieses Netzwerks nicht vorhanden, kann unter Umständen eine Vergabe von zu bearbeitenden Fragestellungen an externe Partner*innen (z. B. Forschungsgruppen und Institute außerhalb des Netzwerks) sinnvoll sein. Darüber

hinaus können entsprechende Kapazitäten auch über ein geeignetes Capacity-Building der Mitarbeitenden im Netzwerk hergestellt werden. Die Forschungspriorisierung sollte demnach eng mit der Initiierung von Forschung verknüpft sein, um zügige Prozesse zu gewährleisten.

7. Weitere Aspekte der Forschungspriorisierung im Kontext einer Pandemie

Über die oben skizzierten Grundprinzipien (siehe Kapitel 4) und konkreten Schritte einer Priorisierung (siehe Kapitel 5) hinaus erscheinen weitere Ansätze notwendig und hilfreich, die zu einer zügigen und effizienten Priorisierung verschiedener Forschungsvorhaben beitragen können. Die im Folgenden skizzierten Strategien bilden Ergebnisse ab, die innerhalb von PREPARED und durch Literaturrecherchen entstanden sind. Für die weitere Ausgestaltung und Implementierung ist vorstellbar, dass die Vorarbeiten aus CEOsys, Ergebnisse aus PREPARED und internationale Ansätze ineinandergreifen und eine Gesamtstrategie der Forschungspriorisierung in pandemischen und sonstigen Notlagen darstellen.

7.1 Fragenkatalog relevanter wissenschaftlicher Fragestellungen aus der COVID-19-Pandemie (Priorisierungsmatrix)

Als Grundlage und zur Beschleunigung der Priorisierung von Fragestellungen im Falle zukünftiger Pandemien erscheint ein Katalog derjenigen Forschungsfragen und Themenbereiche sinnvoll, welche im Zuge der COVID-19-Pandemie als (initial) prioritär identifiziert und behandelt wurden. So ist aus der COVID-19-Pandemie bekannt, dass zu Beginn insbesondere virologische und epidemiologische Fragestellungen relevant waren. Dazu zählen Fragen zur Beschaffenheit des Erregers SARS-CoV-2 (z. B. Infektiosität), zu möglichen Übertragungswegen, zum Schweregrad der hervorgerufenen Symptome und zur Identifizierung von Risikogruppen für eine Infektion in der Bevölkerung. Im Anschluss gewannen Teststrategien und die Güte verschiedener Tests zur Verringerung der Infektionsausbreitung an Bedeutung, bevor im nächsten Schritt das Interesse an der Wirksamkeit von Therapien zur COVID-19-Behandlung, Forschung zu Impfstoffen und der Etablierung von Impfstrategien, den Effekten von Public Health-Maßnahmen (z. B. Reisebeschränkungen) sowie potenziellen psychosozialen Auswirkungen der Pandemie wuchs.

Es ist zu erwarten, dass in einer erneuten Pandemie zu Beginn ähnliche Fragestellungen zu den genannten Themen zu bearbeiten wären, da bei jedem infektiösen Ausbruch zunächst die Art des Erregers und die von ihm ausgehende Bedrohung spezifiziert werden müssen. Durch diesen retrospektiven Blick auf die COVID-19-Pandemie und der daraus resultierenden Festlegung einiger per definitionem bereits priorisierter Fragestellungen könnte sich die Priorisierung von Fragestellungen angesichts zukünftiger Pandemien vor allem auf neu aufkommende Fragen konzentrieren. Wissenschaftliche Ressourcen könnten zu Pandemiebeginn rascher für die Untersuchung bereits

bekannter Fragen eingesetzt, eine Einigung über relevante Studiendesigns erzielt und die Pandemie-Vorbereitung in Deutschland somit verbessert werden.

Konkret wäre ein zweistufiges Vorgehen denkbar. An erster Stelle würde die Bearbeitung bereits prioritärer Fragestellungen und Themen stehen, gefolgt von im Verlauf hinzukommenden Fragestellungen. Dies könnte eine Einsparung von Ressourcen bedeuten, da das zwar standardisierte, jedoch komplexe oben beschriebene Priorisierungsvorgehen nicht sofort in Gänze durchgeführt werden müsste. Vielmehr könnten die ersten Schritte, wie die Bildung eines Priorisierungs-Panels, parallel zur Bearbeitung des Fragenkatalogs durchgeführt werden. Der Fragenkatalog könnte in Form einer Priorisierungsmatrix erstellt werden und in seiner Erstellung einen induktiv-deduktiven Ansatz verfolgen (d. h. Fragestellungen können sowohl aufgrund von bestimmten Ereignissen oder Beobachtungen als auch durch bereits bestehende Annahmen, Zusammenhänge und Theorien aufkommen).

Generell erscheint auch für die Erstellung einer solchen Fragensammlung eine Unterscheidung zwischen verschiedenen Fachdisziplinen und den hier jeweils relevanten spezifischen Fragestellungen sinnvoll, die in die Matrix einfließen können. So könnten beispielsweise über die Einbindung von med. Fachgesellschaften, bekannten Interessengruppen, Kliniker*innen, Public Health-Expert*innen und Wissenschaftler*innen diejenigen Disziplinen in die Erstellung des Fragenkatalogs einbezogen werden, die während der COVID-19-Pandemie besonders aktiv und gefordert waren. Bei der Erstellung der Matrix sollte die Limitierung von Ressourcen bedacht werden. Die Anzahl der Fragen sollte sich daher zwar vorrangig nach der inhaltlichen Notwendigkeit richten, entsprechende Strukturen zur schnellen Bearbeitung im Ernstfall sollten aber zeitlich sichergestellt werden. Hier könnten zusätzlich zu bisher bestehenden Strukturen die in PREPARED geschaffenen Netzwerke, Erfahrungen und Materialien herangezogen werden.

7.2 Etablierung einer repräsentativen Bürger-Kohorte in Deutschland

Zur Unterstützung des oben erwähnten induktiv-deduktiven Ansatzes zur Ermittlung potenziell relevanter wissenschaftlicher Fragestellungen ist auch die dauerhafte Etablierung einer repräsentativen Kohorte in Deutschland sinnvoll. Um die Allgemeinbevölkerung stärker in wissenschaftliche Aktivitäten des NUM einzubeziehen und den Bürger*innen die Möglichkeit zu bieten, Fragestellungen, die auf der individuellen sowie der Bevölkerungsebene als relevant wahrgenommen werden, einzubringen, sollte diese repräsentative Stichprobe regelmäßig in Form von Online-Surveys befragt werden. Auf diese Weise eingegangene Fragestellungen könnten im Anschluss ebenso einen systematischen Priorisierungsprozess nach den oben skizzierten Grundprinzipien durchlaufen.
Ein entsprechendes Konzept wurde im Arbeitspaket 2 „Surveillance, Infektionsprävention und –kontrolle“ von PREPARED entworfen, als Modul in der PREPARED-Infrastruktur eingebaut und kann im entsprechenden Arbeitspapier nachgelesen werden.

Die vorgeschlagene Kohorte könnte bezüglich der Priorisierung eine zusätzliche, aber wesentliche Komponente in der Priorisierungsstrategie darstellen, ein standardisiertes Priorisierungsverfahren allerdings nicht ersetzen.

7.3 Berücksichtigung von Ergebnissen aus Modellierungen (Value of Information Analysis)

Eine weitere Möglichkeit, eine umfassende Strategie zur Priorisierung von Forschungsfragen zu etablieren, stellt die Vernetzung mit dem Forschungsbereich der Modellierungen dar. Prognostische Modelle bzw. Modellierungsstudien haben in der COVID-19-Pandemie eine prominente Rolle eingenommen. Gerade zu Beginn einer Pandemie, wenn noch viele Unklarheiten über das Infektionsgeschehen bestehen und empirische, systematische Forschung erst initiiert und durchgeführt werden muss, bieten sich Modellierungen zur Surveillance des Pandemiegeschehens an.

Für diese Modelle (z. B. zur prognostizierten Bettenauslastung in Krankenhäusern) werden Annahmen benötigt, die in Form von Daten einfließen (z. B. Wie hoch ist die aktuelle Inzidenz in der betrachteten Region? Wie hoch ist die Reproduktionszahl R?). Diese Daten können aus Routinedaten und Surveillancedaten abgeleitet werden. Im Verlauf einer Pandemie verändert sich die Evidenzlage mit der steigenden Anzahl von Studien und damit auch die Möglichkeit, diese Evidenz in entsprechende Modelle einfließen zu lassen und genauere Abschätzungen zu treffen.

Daraus ergibt sich der wechselseitige Nutzen von Modellierungen einerseits und empirischer Forschung andererseits. Modellierungen benötigen bestmögliche Daten, die unter anderem durch empirische Forschung (Evidenz) generiert werden können. Aus den Ergebnissen der Modellierungen ergeben sich wiederum Prognosen und Trends, die weiterführende Fragestellungen und Themen aufwerfen und die ebenfalls Eingang in den Priorisierungsprozess finden müssen. Modellierungen geben also Hinweise darauf, wie sich bestimmte Aspekte der Pandemie entwickeln, und können vor allem auf akuten Forschungs- und Handlungsbedarf hinweisen.

In der intendierten PREPARED Infrastruktur wird dies durch die Kernfunktionalität „MuSE“ umgesetzt. Diese beinhaltet die innovative kombinierte Erfassung von Monitoring und Surveillancedaten – zusammengesetzt aus Infektionssurveillance inkl. Risikofaktoranalyse, Ressourcen und Qualitätsmonitoring sowie Beschäftigtensurveillance. Sie steht in engem Austausch mit dem Modellierungsnetzwerk „MONID“ und ist außerdem eng mit den anderen PREPARED Funktionalitäten verzahnt. Modellierung ist ein wichtiger Bestandteil in der PREPARED Infrastruktur und in diese eingebunden. Sie kann so in verschiedenen Verzahnungen wechselseitig genutzt und befüllt werden.

Auch der Einbezug von „Value of Information“(VoI)-Methoden zur Forschungspriorisierung erscheint nutzbringend (Jackson et al., 2021). VoI-Analysen “quantifizieren den erwarteten Wert von Forschung bei der Verringerung von Entscheidungsunsicherheit“ (Tuffaha, 2021; S. 139). Auf diese Weise erhalten Wissenschaftler*innen sowie andere Interessengruppen Informationen darüber, ob eine Entscheidung auf der Grundlage vorhandener Evidenz getroffen werden kann oder ob zusätzliche Evidenz, beispielsweise durch die Durchführung entsprechender Primärstudien, erforderlich und

sinnvoll ist. Auch für die Priorisierung im Rahmen der Erstellung von systematischen Reviews sind VoI-Ansätze bereits diskutiert worden (Hoomans et al., 2012).

8. Weitere Ressourcen

Es existieren verschiedene Ansätze zur Forschungspriorisierung in der Literatur, die je nach Kontext unterschiedliche Schwerpunkte haben. Für einen ersten Überblick sind hier einige Beispiele gelistet:

- GIN-McMaster Guideline Development Checklist, Topic 2: Priority Setting
- Cochrane Methods Priority Setting, verschiedene Ressourcen (z. B. Checkliste, Guidance-Dokumente, Materialsammlung zu verschiedenen Themen wie Value of Information Analysis)
- James Lind Alliance, Guidebook 2021 (version 10)
- WHO Guidance for staff "A systematic approach for undertaking a research priority-setting exercise", https://apps.who.int/iris/bitstream/handle/10665/334408/9789240009622-eng.pdf?sequence=1&isAllowed=y

9. Literatur

Arbeitsgemeinschaft der Wissenschaftlichen Medizinischen Fachgesellschaften (AWMF)- Ständige Kommission Leitlinien. AWMF-Regelwerk „Leitlinien". 2. Auflage 2020. Verfügbar: http://www.awmf.org/leitlinien/awmf-regelwerk.html (Zugriff am 17.02.2023)

El-Harakeh A, Morsi RZ, Fadlallah R, Bou-Karroum L, Lotfi T, Akl EA. Prioritization approaches in the development of health practice guidelines: a systematic review. *BMC Health Serv Res.* 2019;19(1):692. doi:10.1186/s12913-019-4567-2.

Hoomans T, Seidenfeld J, Basu A, et al. Systematizing the use of Value of Information Analysis in prioritizing sstematic reviews. Rockville (MD): Agency for Healthcare Research and Quality (US); 2012. Verfügbar: https://www.ncbi.nlm.nih.gov/books/NBK107284/ (Zugriff am 17.02.2023)

Jackson C, Johnson R, de Nazelle A, Goel R, de Sá TH, Tainio M, Woodcock J. A guide to value of information methods for prioritising research in health impact modelling. *Epidemiol Methods.* 2021;10(1):20210012. doi:10.1515/em-2021-0012.

Pieper D, Ober P, Dressler C, Schmidt S, Mathes T, Becker M. Effizientere Leitlinienerstellung – eine narrative Übersichtsarbeit [Increasing the efficiency of guideline production: a narrative review]. *Z Evid Fortbild Qual Gesundhwes.* 2019;146:1-6. doi:10.1016/j.zefq.2019.08.004.

Sanabria AJ, Pardo-Hernandez H, Ballesteros M, Canelo-Aybar C, McFarlane E, Niño de Guzman E, Penman K, Posso M, Roqué I Figuls M, Selva A, Vernooij RWM, Alonso-Coello P, Martínez García L; G-I-N Updating Guidelines Working Group and Collaborators. The UpPriority tool was developed to guide the prioritization of clinical guideline questions for updating. *J Clin Epidemiol.* 2020;126:80-92. doi:10.1016/j.jclinepi.2020.06.018.

Tuffaha H. Value of Information Analysis: Are we there yet? *Pharmacoecon Open.* 2021;5(2):139-141. doi:10.1007/s41669-020-00227-6.

10. Anhang

Tabelle 1. Template zu Bewertungskriterien aus CEOsys für die Priorisierung von Versorgungsfragen

**Dimensionen und Kriterien**		**Fragestellung**<br>(z. B. Wirksamkeit von Interventionen zur Förderung von psychischer Gesundheit, Resilienz oder Stressmanagement bei Gesundheitspersonal angesichts der COVID-19 Pandemie)
**I. Krankheitslast**		**Bezogen auf die o. g. PICO-Frage, wie hoch ist/sind…**
1.	Individuelle Krankheitslast	…die individuelle Krankheitslast?
2.	„*public*“ Krankheitslast	…die bevölkerungsbezogene Krankheitslast (health burden)?
3.	Ökonomische Bedeutung	…die negativen ökonomischen Folgen aus volkswirtschaftlicher Perspektive (economic burden)?
**II. Versorgung**		
4.	Varianzen/Unsicherheit	…die Versorgungsvarianz ohne medizinische/plausible Begründung?
5.	Koordinationsbedarf	…der interdisziplinäre, interprofessionelle oder intersektorale Koordinationsbedarf?
6.	Ethische/soziale Aspekte	…das Risiko für Versorgungsungerechtigkeit (*Equity*) und Zugangsbarrieren?
**III. Forschungsdynamik**		
7.	Evidenz/*Researchability*	…das Aufkommen geplanter, laufender oder kürzlich abgeschlossener Primärstudien dazu?
**IV. Konsequenzen**		
8.	Optimierungspotential/ Entscheidungsrelevanz	…das Potenzial, durch eine Aktualisierung von Evidenzsynthesen und Leitlinienempfehlungen die Versorgung des Gesundheitsproblems (in Deutschland) zu optimieren?
9.	Implementation	…die Machbarkeit der Intervention/Verhaltensänderung unter Berücksichtigung auch von Ressourcen und Widerständen?
10.	Gesundheitssystem	…die Auswirkungen auf das Gesundheitswesen (Leistungsfähigkeit, Betten-, Intensiv-, Personalkapazitäten)?
11.	Gesellschaft	…die Auswirkungen auf die Gesamtgesellschaft (z. B. Stabilität, Produktivität, Sicherheit)?
**V. Relevanz im Fachgebiet**		
12.	Themenfeld-Bewertung	…die Bedeutung innerhalb Ihres Fachgebiets?
